# Advances in the sample pretreatment for determination of microplastics in riverbed sediments by Raman spectroscopy

**DOI:** 10.1007/s11356-026-38231-8

**Published:** 2026-09-25

**Authors:** Andrea Dali, Mirco Mancini, Daniela Santianni, Luca Solari, Maurizio Becucci

**Affiliations:** 1 Department of Chemistry “Ugo Schiff” (DICUS), University of Florence, Via Della Lastruccia 3-13, 50019 Sesto Fiorentino, FI Italy; 2 Department of Civil and Environmental Engineering, University of Florence, Via Santa Marta 3, 50139 Florence, Italy; 3 Publiacqua S.P.A., Via Villamagna 90C, 50126 Florence, Italy; 4 INSTM, Research Unit of Florence, Via Della Lastruccia 3, 50019 Sesto Fiorentino, FI Italy

**Keywords:** Microplastic extraction, Microplastic quantification, Raman spectroscopy, Arno River, River sediment, Sample pretreatment

## Abstract

Microplastics (MPs) represent an important global environmental threat, and, among the different environmental compartments, the aquatic systems are particularly impacted by the MPs problem. River sediments act as sinks and as major pathways for MPs transportation to marine systems. Therefore, there is an increasing need for comprehensive, basin-scale monitoring of MPs in river sediments to elucidate their spatial and temporal accumulation patterns, providing data essential for risk assessment and the development of effective mitigation strategies by stakeholders. MPs extraction from complex environmental samples remains challenging as the presence of organic and inorganic matter often interferes with the analytical identification. To address this gap, we optimized a low-cost, high-throughput sample preparation protocol specifically designed for analysis of large riverine sediment sets and accurate Raman identification. The protocol was validated across a wide cross-section of sediment matrices, from the Arno River (Pisa and Florence, Italy) with different grain size distribution and organic matter concentrations. This validation, with both spiked matrices and real environmental sediments, demonstrates a workflow that is tailored to handle sediments rich in organic and inorganic matter while enabling accurate identification of MPs.

**Supplementary Information:**

The online version contains supplementary material available at https://doi.org/10.1007/s11356-026-38231-8.

## Introduction

Global plastic production has increased dramatically over the past decades, rising from approximately 2 million metric tons in 1950 to 390.7 million metric tons in 2021 (Plastics Europe 2022). This dramatic growth is largely driven by the low cost, versatility, and high durability of plastic materials (Thompson et al. 2009). However, since most plastics are non-biodegradable, their widespread production has resulted in the accumulation of vast amounts of persistent waste, much of which is inadequately managed and eventually released into the environment (Geyer et al. 2017; Cottom et al. 2024). Macroplastics (MAPs, dimensions > 25 mm) released into the environment undergo progressive fragmentation due to physical weathering and photodegradation, leading to the formation of mesoplastics (MEPs, dimensions between 5 and 25 mm) and microplastics (MPs, dimensions between 1 µm and 5 mm) (Julienne et al. 2019; Andrady 2022; Rambacher et al. 2023). MPs are typically defined as solid polymer-containing particles ranging from 1 µm to 5 mm in size, excluding (bio)degradable polymers (European Chemicals Agency (ECHA) 2019). Based on their origin, MPs are classified as primary, intentionally manufactured at microscopic sizes, or secondary, which originate from the fragmentation of larger plastic debris (Emberson-Marl et al. 2023; Nikhil et al. 2024). Consequently, MPs in natural environments exhibit considerable diversity in polymer types, shapes, color and particle sizes (Kooi et al. 2021).

Among all environmental compartments, aquatic systems are particularly impacted by plastic pollution (Schmidt et al. 2017a, b; Lebreton et al. 2022). It is estimated that, in 2015 alone, between 4.8 and 12.7 million metric tons of plastic waste entered the oceans from terrestrial sources (Jambeck et al. 2015). Freshwater environments, including rivers, lakes, streams, wetlands, and groundwater, serve both as sinks and as major pathways for MPs transportation to marine systems (Dris et al. 2015; D’Avignon et al. 2022; Dalvand and Hamidian 2023; Dikareva and Simon 2024; Xu et al. 2024). Once in the aquatic environment, MPs can adversely affect organisms that ingest plastic debris, inducing physical and chemical stress that impacts feeding behavior, energy metabolism, and reproductive success (Cole et al. 2013, 2019; Gardon et al. 2018; Covernton et al. 2021; Jewett et al. 2022). Moreover, MPs can act as vectors for toxic substances, including persistent organic pollutants (POPs) and heavy metals, which bioaccumulate in the food chain, posing risks to aquatic organisms and ultimately humans (Ding et al. 2022; Okoye et al. 2022; Tumwesigye et al. 2023). These considerations underscore the importance of large-scale mapping of MPs to determine their spatial and temporal accumulation patterns in riverine sediments and to enable accurate quantification necessary for environmental risk assessment.

However, collecting, handling, identifying, and characterizing MPs from environmental samples remains challenging due to their small size and the high diversity in shape, color, density, and physical–chemical properties. This is especially true in aquatic environments, where particle buoyancy, biofouling processes, aggregation with organic and inorganic matter, and continuous transport driven by currents and turbulence significantly influence their distribution, transformation, and detectability. Currently, the primary methods for MP identification rely on vibrational spectroscopy, namely Raman spectroscopy (RS) and Fourier-transform infrared (FT-IR) spectroscopy, which are favored due to their non-destructive nature and ability to discriminate between plastic and natural particles (Vianello et al. 2018; Chakraborty et al. 2023; Monnanni et al. 2024). Simpler techniques based on selective staining, such as Nile Red, are less specific and can interfere with spectroscopic analyses due to fluorescence (Stanton et al. 2019; Sancataldo et al. 2020; Prasad et al. 2024). Particularly, microRaman is a fast, non-destructive and non-contact method capable of providing both chemical and morphological information (Nava and Leoni 2021; Chakraborty et al. 2023; Kumar et al. 2026). Moreover, RS can also identify pigments, dyes and other additives (Casadio et al. 2010; Lin and Chang 2022; Kumar et al. 2026). Its popularity has increased with the availability of efficient, cost-effective laser sources and detectors, and its use of visible radiation allows it to surpass the 10-µm diffraction limit of micro-FTIR spectroscopy (Chakraborty et al. 2023).

Although MPs have been studied extensively in diverse water bodies and sediments (Scopetani et al. 2020; Liu et al. 2020; Jiao et al. 2021; Chakraborty et al. 2023; Simon-Sánchez et al. 2024), there is still no standardized, robust protocol for sampling and analysis (Hermsen et al. 2018; Kumar et al. 2026) in complex matrices. As a result, field studies often produce non-comparable results. In addition to accuracy, methods must be rapid and cost-effective to allow for extensive sampling campaigns over large spatial and temporal scales. Sample preparation is particularly critical for sediments, which are important sinks for MPs and key indicators of long-term accumulation (Hidalgo-Ruz et al. 2012; Liu et al. 2020). Sediments are challenging due to their intrinsic site-specific composition, being a mixture of light organic matter and fine inorganic particles, which can trap MPs and hinder recovery. Multi-step extraction protocols are often required, including oxidation, density separation, enzymatic digestion, and filtration (Hidalgo-Ruz et al. 2012; Simon-Sánchez et al. 2024; Kumar et al. 2026). However, while effective, these are time-consuming and expensive. Alternative faster approaches, such as electrostatic or magnetic separation and oil-based extraction methods, have been proposed (Felsing et al. 2018; Grbic et al. 2019; Scopetani et al. 2020), yet these may be incompatible with RS due to residual materials generating fluorescence or sample handling difficulties. Also, other two-phase system-based approaches have been recently proposed as alternative MPs extraction methods from seawater, beach sand, and marine sediments. These separation techniques successfully isolate MPs by effectively partitioning them away from common natural biopolymer interferents (Liu et al. 2022, 2024; Chang et al. 2023).

To date, to the best of our knowledge, a rapid, low-cost, and sediment-specific extraction protocol compatible with microRaman analysis has not yet been reported. However, high-throughput methods are mandatory to have large-scale monitoring programs of the MPs concentration in riverine systems (i.e., to evaluate the temporal dynamics of MP pollution). To address this gap, we optimized a sample preparation protocol specifically for riverine sediments, addressing the limitations of existing methods. Preliminary experiments were conducted using spiked test sediments and environmental samples from the Arno River (at locations in Pisa and Florence, Italy), demonstrating a workflow that is reasonably fast, inexpensive, and tailored for sediments rich in organic and inorganic matter while enabling accurate RS analysis of MPs.

## Materials and methods

### Sediment sampling and characterization

Environmental sediment samples were obtained through the sampling of riverbed sediments from the Arno River along the stretch that flows through the cities of Florence (five samples, sampling location N1) and Pisa (one sample, sampling location N2) (Italy) in June 2025 (Figure S1). This river was selected as suitable for three main reasons: (i) the availability of previous studies investigating MP concentrations both in the water column (Monnanni et al. 2024) and in bottom sediments (Rimondi et al. 2022; Ghinassi et al. 2023) in its nearby areas, as well as in treated wastewater discharged into it by local wastewater treatment plants (Becucci et al. 2022), albeit using sampling and analytical methodologies different from those proposed in this study; (ii) the strong presence of industrial and tourism-related activities characterizing the area, potentially generating a wide variety of polymer types detectable in the aquatic environment; (iii) the presence of riverbed sediments with highly heterogeneous grain size distributions, ranging from silts and clays to coarse sands and fine gravel (Figure S2). Additionally, sediment sampling was conducted during the summer season, under mean discharge conditions of the order of 10–20 m^3^ s⁻^1^ (Peruzzi et al. 2019), and in the absence of rainfall during the 48 h preceding sampling. Under these low-flow conditions and in areas characterized by slow or nearly stagnant flow, a maximization of organic matter accumulation in bottom sediments is generally observed, deriving both from the deposition of allochthonous plant detritus and from locally produced biomass (Vannote et al. 1980; Naiman and Décamps 1997).

Each of the sampling points was identified in advance using cartography (Google Maps) and a portable GPS device (Garmin GPSMAP 66i) for field-based georeferencing by testing different distances from the riverbank, varying water depths, and sites characterized by different sediment grain size distributions. The samples were collected by using a stainless steel Van Veen gravity corer, capable of sampling an area of 400 cm^2^ to a depth of approximately 15 cm (equivalent to a sediment volume of 6 dm^3^). The samples were immediately stirred and transferred into a 250-ml glass container and subsequently stored in a refrigerator, away from light, to prevent alteration of the sediment matrix due to algal growth.

Before laboratory analysis, the sediment samples were placed in an aluminum container and dried in an oven at 90 °C for 24 h, weighed, and sieved using stainless steel sieves with mesh sizes between 5 and 1 mm. All materials above 1 mm, occasionally found, were retained for separate inspection. This preliminary sieving process facilitates the visual identification of larger MPs (≥ 1 mm) and removes coarser organic components (e.g., twigs, leaves, shells) typically present in streambed sediments. The removed coarser organic components were washed with filtered demineralized water and transferred to the sample ready for the extraction methods (see Chapter 2.3 *Extraction methods*) to release the potentially trapped MPs.

In parallel, a subsample of the dried sediment was used to determine grain size distribution by combining conventional sieve analysis, performed with a standard ASTM sieve stack for the coarse fraction (d_50_ > 500 µm), with laser scattering particle size analysis (LISST-PORTABLE XR, SN 709, Sequoia®) for smaller fraction (d_50_ < 500 µm).

### Organic carbon determination

To quantify the organic fraction interfering with the MPs determination, the total amount of organic carbon was measured on the sediment fraction passing through the preliminary 1–5-mm sieve. By excluding the coarser organic debris, this method evaluated only the organic load that could potentially hide MPs and affect the efficiency of the MPs extraction protocol. The organic carbon of each sample was determined using an elemental analyzer (FLASH EA 1112, Thermo Fisher Scientific, Milan, Italy).

### Extraction methods

**Fig. 1:**
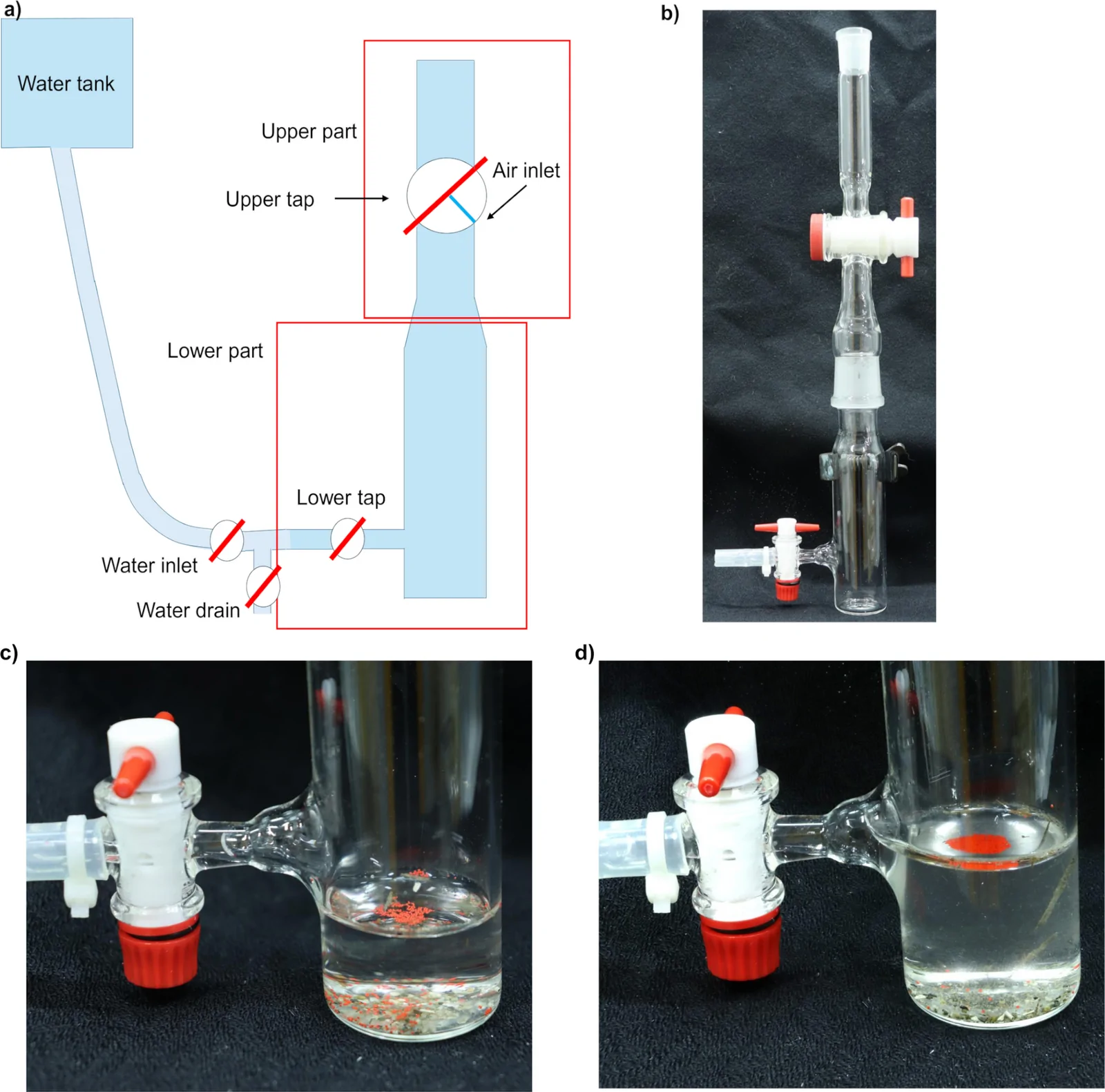
Custom-made glass separator for microplastics (MPs) extraction. **a** Schematic representation of the device, consisting of separable upper and lower parts, each part with an independent tap (namely upper and lower, respectively). The system is connected to a filtered demineralized water reservoir and includes an air inlet, and a water drain and inlet port. **b** Photograph of the assembled glass separator. **c** Close-up photograph of a spiked sediment sample suspended in water. **d** Photograph of the same sample after addition of cyclohexane and mixing. MPs selectively move into the upper non-polar cyclohexane phase, while residual organic matter remains in the lower water phase

For each sample, approximately 30 g of pre-sieved sediments were put in a glass beaker together with 30 ml of filtered demineralized water. The filtered demineralized water was obtained by filtering demineralized water using Whatman filter papers (grade 2, 8 μm pore diameter) to remove any MPs or other solid contaminants present in the water. The efficiency of MP removal from water was tested by preparing blank samples (see below). The samples were first sonicated for 5 min at 35 kHz (Astrason, Farmingdale, USA) to expose the MPs and then oxidized using a Fenton protocol slightly modified from that reported in Refs. (Qin et al. 2015; Scopetani et al. 2020). In particular, we added in the beaker the Fenton reagents (60 ml of 30% H_2_O_2_ (Carlo Erba); 6 ml of a 2 mM solution of FeSO_4_^.^7H_2_O (Sigma Aldrich); 6 ml of a 2 mM solution of acetic acid (Sigma Aldrich)). The reaction was kept at 35 °C for 1 week or until the reaction stops, i.e., no more foam was formed even upon further H_2_O_2_ addition (Liu et al. 2019; Simon-Sánchez et al. 2024). Then, the samples were filtered (Whatman filter papers, grade 2, 8 μm pore diameter) to remove the separate solid from the reagent solutions. The material remaining on the filter was then recovered by backwashing it and density separated using a saturated NaCl aqueous solution. Visual inspection under a UV lamp was performed to ensure the complete absence of residual MPs on the filter. The saturated NaCl solution was prepared by dissolving NaCl in water until saturation was reached (338 g/l) and then filtered using Whatman filter papers (8 μm pore diameter) to remove MPs or other solid contaminants. The sample was first shaken for 1 min and then left to settle for 4 h (Hidalgo-Ruz et al. 2012). After 4 h, the floating fraction was recovered and solids were collected by filtration (Whatman filter, 8-μm pore diameter) and washed with filtered demineralized water to remove the salt. Since Fenton oxidation leaves some organic residues in the samples a subsequent separation using a custom-made glass separator was implemented. In the glass separator (Fig. 1, panels a and b) the filtered materials are in contact with a biphasic liquid system made of water and cyclohexane. As shown in Fig. 1, panels c and d, MPs are preferentially suspended in the organic phase, while residuals of vegetables are more likely to remain in contact with water. We operate as follows, under the fume hood: 2 ml of cyclohexane (Carlo Erba) were added into the lower part of the separator, then the sample recovered from the filter was added using about 5 ml of water and 0.1 ml of a 3 mM solution of sodium dodecyl sulfate—SDS (Sigma Aldrich). The surfactant (i.e., the SDS) was used to minimize the adhesive forces between MPs and sediment matrix and therefore, to increase the efficiency of the extraction. Following, enough filtered demineralized water was added to fill up about ⅔ of the lower part volume. The water was added taking care to generate enough turbulence to mix the sample. After this step, the upper part of the separator was mounted and, from the bottom valve, filtered demineralized water was added to push the organic phase, carrying MPs, upwards and overpass the top valve. Once the desired level was reached in the upper section, the top valve and the water inlet were closed in sequence; then, the drain valve was opened and finally, air (from the air inlet) was allowed to enter making water (in the glass separator) to drain from the bottom valve. Once the liquid level drops below the lower section, the upper part of the glassware was removed and the sample recovered in a clean beaker. The particles possibly trapped in the glass joint (i.e., between the upper and lower parts) were remixed, using water, in the residual sample. The process, including SDS addition, was repeated three times to improve the MP recovery. Finally, the sample collected was filtered using a cellulose nitrate filter with 5 µm pore size (Sartorius Stedim Biotech GmnH - Gesellschaft mit beschränkter Haftung) and transferred into a clean glass Petri dish. Still, operating under the fume hood, after solvent evaporation, samples were stored in a clean glass Petri dish (with cover) at room temperature while waiting for the RS investigation. The cellulose nitrate filter was used for the final filtration of the sample as it does not interfere with the Raman characterization of MPs, and it has a low price.

### Method validation for MPs determination

The efficiency and capability of the proposed extraction method to separate the MPs from complex matrices was evaluated through a two-step validation process on real sediment samples from the Arno River. Each of these sediment samples was divided into two 30 g aliquots. The first aliquot was spiked with reference particles and, after applying the extraction protocol, the recovered MPs were counted to evaluate the extraction efficiency within a real environmental matrix. Recovery efficiency was calculated as the recovery fraction (the fraction of particles successfully retrieved after the extraction process). The second aliquot was processed to quantify the actual environmental MPs concentration, thereby demonstrating the efficiency of the method in isolating MPs from other inorganic and organic parts present in the sample; identifying and quantifying different types of MPs by using Raman spectroscopies technique.

The reference MPs (Sigma Aldrich) made of different polymers were used to cover a range of densities (Table S1): polyethylene (PE, 0.98 g/cm^3^), acrylonitrile butadiene styrene (ABS, 1.01 g/cm^3^), polyvinyl chloride (PVC, 1.084 g/cm^3^), polyamide (PA, 1.14 g/cm^3^) and polyethylene terephthalate (PET, 1.30 g/cm^3^). Moreover, to assess the influence of particle morphology on recovery rate, other parameters were varied (Figure S3 and Table S1): (i) the particle size (nominal diameter, D_n_, ranged from 388 μm to 1.7 mm); (ii) the particle shape through the aspect ratio (AS) ranged from 1.02 to 5.26. The values of D_n_ and AS are obtained as the average of the value found on 30 MPs fragments between the total MPs used in the test. For each polymer in Table S1 the wettability is also reported, expressed as the contact angle with water. The values were taken from the literature for pristine materials. We made the effort to measure the contact angle for cyclohexane on the different materials under investigation. For many polymers an almost complete spreading was observed, corresponding to a very small contact angle (< 5°, not precisely determined), demonstrating the very good affinity of the polymers with this solvent. Only for PET, a polymer containing polar groups, we were able to directly measure the contact angle with both water and cyclohexane (Figure S4). Measurements were performed using 10 µL droplets dispensed using a precision pipette. The angles were determined using the ContactAngle tool in ImageJ, and each value represents the average of five independent measurements for each liquid. The measured water contact angle on PET (78° ± 0.7°) is in good alignment with reference data in the literature (81°) (Ellison and Zisman 1954) while the contact angle for cyclohexane was much lower (19° ± 3.7°), demonstrating a larger affinity for the organic solvent compared to water even for this material.

### Measures adopted to minimize cross- and external contamination

To avoid problems of cross- and external contamination, sampling was conducted using plastic-free equipment and the experiment was performed in a controlled and clean environment. All glassware was rinsed at least three times with filtered demineralized water and detergent prior to use, wiped using lint-free wipers, and immediately covered with aluminum foil and watch glasses when not in active use (Dris et al. 2018; Brander et al. 2020; Becucci et al. 2022; Kumar et al. 2026). Moreover, the number of samples transferred between different containers was minimized. To avoid self-contamination during sampling, pre-treatment and analysis of samples laboratory staff used only cotton clothes, particle-free nitrile gloves and, moreover fleece clothing and plastic items were strictly avoided (Hermsen et al. 2018; Witzig et al. 2020; Becucci et al. 2022; Kumar et al. 2026). As the cellulose nitrate filters can be contaminated even before their first use, they were blown with a jet of filtered compressed air before sample filtration. Further, two sets of three blank samples were prepared together with the samples to check for contamination. No MPs larger than 20 µm (the smaller dimension we target in our analysis) were found in any of the blanks and, therefore, no mathematical blank correction or subtraction protocol was necessary.

### Hyperspectral imaging and Raman analysis, identification, and quantification

Preliminarily, RGB and fluorescence (370 nm excitation) images of the filter holding the sample were collected, using an ordinary digital camera Canon EOS250D with UV-filtered macro-optics, to support the operations under the microscope. As described in Becucci et al. 2022, these images were combined and processed using ImageJ software to highlight particles present on the filter (for an example, see Fig. 5). This screening analysis identified larger dimension MP candidates.

Raman analysis was conducted using a microRaman spectrometer RM2000 (Renishaw, Wotton-under-Edge, UK) equipped with Olympus microscope objective (5x/0.10 N.A., 20x/0.40 N.A. and 50x/0.80 N.A.) and a 785-nm diode. Samples were put on computer-controlled motorized stage (accuracy 1 µm) and, using a ×20 microscope objective, all MP candidates were investigated. Moreover, the entire filters were scanned using a ×5 microscope objective to detect any additional particulates with dimensions above 20 μm. This dimensional limit was set given the natural background particles found in the samples and the filtration step with stainless grid. For each selected particle, both picture (with calibrated axes) and Raman spectrum were recorded. The Raman spectra wavenumber scale was routinely calibrated on the 520 cm^−1^ first-order Raman band of bulk silicon: wavenumber accuracy along the full spectrum is 2 cm^−1^ and the spectral resolution is 5 cm^−1^.

The chemical composition of the particles was obtained based on their Raman spectrum upon elaboration on the web by using the open-source program OpenSpecy and its associated libraries (https://www.openanalysis.org/openspecy/) (Cowger et al. 2025). All raw Raman spectra underwent a standardized preprocessing protocol within the software, consisting of baseline correction and min–max intensity normalization. For the identification, a dual-verification approach was employed: (i) the correlation ranking generated by OpenSpecy was utilized as an initial screening tool; (ii) a visual inspection was conducted by an operator to confirm the overlap of the main vibrational bands between the experimental and reference spectra.

Since H_2_O_2_ oxidation can potentially degrade MPs (Kumar et al. 2026), a stability test was performed on purchased MPs (see Chapter 2.4 *Method validation for MPs determination*). It was verified that the Raman spectra recorded both before and after the extraction protocols were identical (data not shown). Furthermore, no changes in the dimensions, shape and morphology of the MPs were observed, confirming their integrity after the process (data not shown).

## Results and discussion

### Development of the methods

**Fig. 2:**
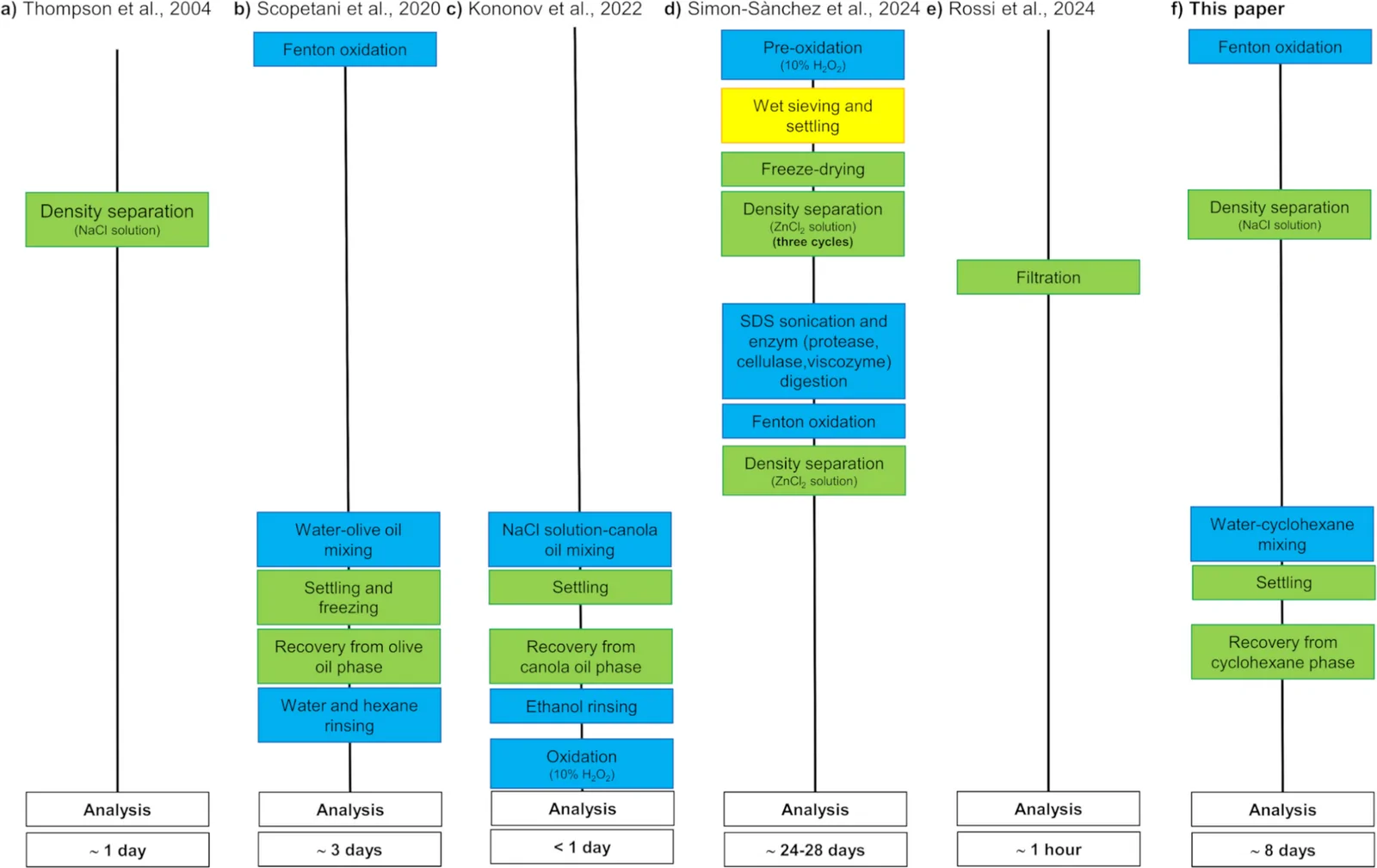
Overview of sample pre-treatment protocols for MP analysis in soils and sediments. Schematic comparison of methodologies proposed, ordered chronologically, by **a** Ref. Hidalgo-Ruz et al. (2012), **b** Ref. Scopetani et al. (2020), **c** Ref. Kononov et al. (2022), **d** Ref. Simon-Sánchez et al. (2024), **e** Ref. Rossi et al. (2024) and **f** this paper. The blue, yellow, and green colors indicated the chemical, mechanical, and physical process, respectively. Estimated processing times, indicated in figure, does not consider sampling, drying and weighting steps. The filtration steps are not reported in the figure; exception is made for protocol **e** where it was the only preparation step performed

The analysis of MPs in river sediments is essential, as fluvial ecosystems are recognized as major drivers of microparticles toward seas, including highly impacted basins such as the Mediterranean Sea (Cantasano 2022). Moreover, sediments act as a significant sink for MPs (Weber and Lechthaler 2021; Rohais et al. 2024; Mancini et al. 2025). Protocols commonly used for water samples with low contents of total suspended solids, such as drinking water or clean fluvial water, are inadequate for sediment matrices. In fact, the recovery and identification of MPs from river sediments are significantly more complicated due to the abundance of organic and inorganic material, which interferes with the analysis and cannot be easily removed by density separation alone. Several sediment-preparation protocols are reported in the literature (Hidalgo-Ruz et al. 2012) and generally rely on the method proposed by Thompson and co-worker (Thompson et al. 2004). These protocols make use of sieving, density separation and filtration and were applied principally for marine and beach sediments or, in general, sediments characterized by low organic contents (Hidalgo-Ruz et al. 2012) (Fig. 2a). However, when dealing with organic-rich river sediments, these basic steps are insufficient. Consequently, for such matrices, chemical digestion and oxidation are required to eliminate the organic contaminants (such as proteins, carbohydrates, and fats) that commonly interfere with subsequent steps for the MPs chemical identification. Oxidation is generally carried out through Fenton protocols, whereas complete removal of the organic material requires further efforts, such as enzymatic digestion using different enzymes like protease or cellulase. For instance, a multi-step protocol, requiring approximately 24 days, was recently applied to sediments from the Limfjord River in Denmark (Simon-Sánchez et al. 2024) (Fig. 2d).

Recently, a method was also proposed to analyze MPs in marine sediments without pretreatments (Fig. 2e) (Rossi et al. 2024). But this protocol, as admitted by the authors, investigates a small amount of sediments (1 g) on each analysis and it has some disadvantages such as the need for experienced users and high-quality and high-cost instruments not easily available in laboratories.

Alternative simpler high-throughput techniques, such as dye staining methods (e.g., using Nile Red—NR—and fluorescence microscopy), are applicable for clean matrices like bottled water, but are not suitable for organic-rich samples. On one hand, organic-rich samples leave residual organic matter or cellular lipids that co-stain with NR, leading to potential false positives and interfering with the accurate screening of MPs (Chatterjee et al. 2023; Villa et al. 2025). On the other hand, it has already been proved that the oxidative step can lead to the presence of oxidized groups on the MPs surface resulting in a shifted fluorescence emission from the staining dye (Idehara et al. 2025). Furthermore, residual natural organic matter leads to an intrinsic fluorescence background in the RS experiments. The problem of fluorescence background in Raman spectra can be handled by time-gated (Kotula et al. 2022) or shifted excitation methods (Xue et al. 2024). However, these approaches require specialized and expensive instruments. As a representative example, Figure S5 showed the significantly increased noise level of the Raman spectrum (baseline subtracted) measured on NR-stained ABS particles with respect to that measured on pristine ABS due to the shot noise associated to the high level of fluorescence background. A similar effect was observed also for other polymers (data not shown) as the fluorescence interference is not directly associated to the polymer chemical nature but to the dyes possibly present (either added in the sample preparation step as staining or used during the polymer production). While this background is already problematic on this stained reference MPs, it makes the identification of smaller environmental MPs practically impossible.

Other methods reported in literature need particular instrumentation to separate soil and aquatic sediments from MPs, such as magnetic separation, pressurized liquid extraction, and therefore are not ideal to be applied to monitor high number of samples with operative low cost. Another possibility, not relying on particular instrumentation, is represented by the method based on oil leaching. Here the hydrophobic affinity between oil and MPs makes the MPs migrate in the oil phase where they can be extracted (Huang et al. 2024). However, traditional funnels can be clogged by solid samples, requiring more advanced systems. For example, Kononov and coworkers used a combination of an extraction method in canola oil and a density separation (NaCl) together with H_2_O_2_ oxidation after extraction (Fig. 2c) (Kononov et al. 2022). This method was then improved by Li and coworkers by optimization of the oil–water ratio and by creating microemulsions (Li et al. 2023). However, these were applied on commercial products (toothpaste and tea) or simple aqueous samples (seawater or soil supernatant). Differently, in 2020, Scopetani and coworkers developed a non-density based, inexpensive, simple and safe extraction method for sediment preparation (Scopetani et al. 2020). Their method, optimized for FT-IR analysis, achieved high recovery rate by combining the Fenton oxidation with an oil-assisted extraction step (Fig. 2b). While faster, economical and capable of recovering high-density polymers, we found that in our organic-rich samples, organic debris tended to collect at the water–oil interface making their removal quite difficult, trapping MPs and hindering recovery. Moreover, also after water and hexane rinsing, the olive oil residues on the filter were fluorescent and, therefore, prevented the RS identification of MPs (for example see Figure S6). This highlights a significant limitation of oil-assisted methods coupled with RS. Similarly, recent advances in two-phase system-based approaches, such as aqueous two-phase systems and advanced surfactant-driven biphasic extraction, have achieved efficient MPs isolation from substrates like seawater, beach sand, and marine sediments by removing natural biopolymers like chitin, lignin, and cellulose (Liu et al. 2022, 2024; Chang et al. 2023). However, the application of these protocols to highly cohesive, organic-rich riverbed sediments showing severe particle aggregation is still to be proved.

To enable MP identification by RS, we modified the original *olive oil method* by replacing olive oil with cyclohexane. Cyclohexane was selected over other non-polar solvents because it yields a good MP recovery (Fig. 1 and see Chapter 3.2 *Tested recovery of MPs*), does not stick to the glassware, does not increase the fluorescence background of both filters and MPs and it is easily removed by evaporation. Cyclohexane is a solvent with very high optical transparency as its electronic absorption spectrum starts around 200–210 nm. Therefore, with visible light excitation there is no optical absorption and fluorescence emission. This is especially true for Cyclohexane Spectroscopic Grade; however also the solvent at Reagent grade could be satisfactorily used for this application as its major impurity is benzene and that will affect only its properties for ultraviolet excitation at wavelength below 270 nm, very far off the visible range. Its optical transparency is related to its structure and electronic properties, cyclohexane being a saturated, symmetrical aliphatic hydrocarbon, and as such it does not contain conjugated electrons or heteroatoms. Cyclohexane, due to the above-described properties, even if present into the sample (unlikely, due to its high volatility) does not generate its own fluorescence signal (background noise) in the near UV-Visible range maximizing instrument sensitivity even for very weak signals in Raman scattering experiments. Furthermore, unlike solvents containing heavy atoms (such as dichloromethane or chloroform) or reactive functional groups, cyclohexane is chemically inert under analytical conditions. Therefore, free radicals (possibly present in the sample, from the previous Fenton oxidation step) are less prone to induce solvent chemical degradation which could lead to fluorescing residues. Although cyclohexane from a safety, health and environmental point of view is ranked as “problematic” (i.e., it can be used in the lab but its implementation in the production scale will require specific measures) (Alder et al. 2016; Prat et al. 2016), with a known aquatic toxicity (acute and chronic), its impact in our protocol is mitigated by the small volume of solvent used for each sample (total 6 ml). Additionally, the recovery of the frozen organic phase in the original method proved to be challenging given the residual organic material still present in our samples after the oxidation step. A custom-made glass extractor was designed to overcome these difficulties (Fig. 1) and to avoid freezing the sample as in Ref. (Scopetani et al. 2020). Crucially, this custom-made separator was also designed to significantly minimize chemical cyclohexane waste. Finally, the specific properties of our samples (i.e., the high content of mineral sediments and natural organic residues) led us to introduce a preliminary density separation step in the protocol (Fig. 2f). The proposed protocol was tested in the laboratory to evaluate its efficiency and suitability for RS based identification.

### Tested recovery of MPs

**Fig. 3:**
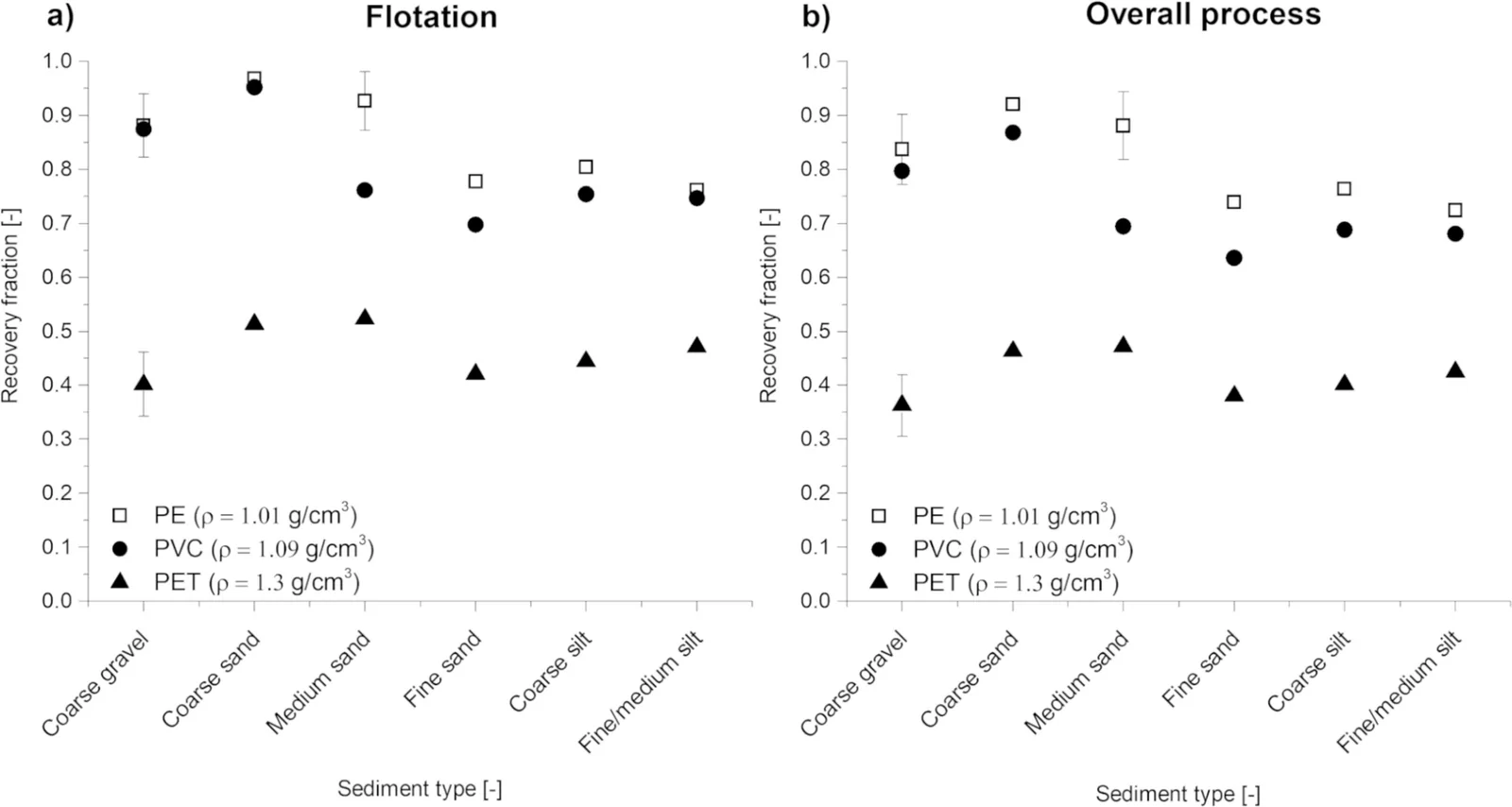
Microplastic recovery fraction for polyethylene (PE), polyvinyl chloride (PVC) and polyethylene terephthalate (PET). Results are shown for **a** the flotation process and **b** the overall process tested for different types of sediment. Also, densities of the polymers (g/cm^3^) are indicated. Numerical values are reported in Table S2

To evaluate the efficiency of the overall proposed protocol, we tested the method on different spiked environmental samples where an exact number of MPs was mixed with real sediment samples collected from the Arno River in the city center of Florence and Pisa (see Chapter 2. *Materials and methods*). These samples exhibit different grain size distributions, ranging from fine/medium silts (D_50_ = 0.01 mm) and coarse silts (D_50_ = 0.24 mm) to coarse gravel (D_50_ = 39.98 mm), together with different organic carbon contents (0.14–2.2%). Therefore, they represent a cross-section of different riverine environments that can be found in analyzing real riverine sediments. Efficiency was measured as the MP recovery fraction, expressed as the ratio of recovered to initially added MPs in the testing samples. In the flotation step (Fig. 3a), the overall efficiency was 0.70 ± 0.19 with significant variations based on polymer density. As expected, higher recovery was found for the low (PE: 0.85 ± 0.08) and medium (PVC: 0.79 ± 0.09) density polymers. For high-density polymers (PET), the efficiency dropped at 0.46 ± 0.06. Looking then at the overall process (Fig. 3b), this trend was maintained with an overall efficiency of 0.65 ± 0.19 and an efficiency of 0.82 ± 0.08, 0.72 ± 0.09 and 0.42 ± 0.06 for the low, medium and high-density polymers, respectively. It is evident that the limiting factor is the flotation step and, in particular, the 1.2 g/cm^3^ density of the saturated NaCl aqueous solution.

In this flotation agent, high-density polymers (like PET) float only thanks to the presence of air microbubbles or other physical factors (e.g., stabilizing surface forces) (Pita and Castilho 2020). While higher density salt solutions (such as NaI or ZnCl_2_) could help to overcome this problem, they were excluded due to their higher costs, toxicity and problematic handling of large volumes of hazardous reactants (Kedzierski et al. 2017; Rodrigues et al. 2020; Huang et al. 2024; Jefroy et al. 2026). In detail, ZnCl_2_ is highly ecotoxic (Huang et al. 2024) and its corrosiveness can affect flotation efficiency (Elkhatib and Oyanedel-Craver 2020; Huang et al. 2024) while NaI requires energy-intensive recycling procedures to be economically viable (Kedzierski et al. 2017). In fact, NaCl is still one of the most used salts (Hidalgo-Ruz et al. 2012; Miller et al. 2017). Consequently, while the proposed method yields highly quantitative recovery rates for low and medium-density polymers, it should be considered *semi-quantitative* for high-density polymers (density above 1.2 g/cm^3^). We accepted this as a necessary trade-off to maintain a cost-effective and simple implementation protocol. This compromise is also justified by the fact that the majority of MPs found in aquatic environments are characterized by densities below 1.2 g/cm^3^ (Kooi and Koelmans 2019). On the overall global plastic production only approximately 13% are polymers with densities higher than this threshold (Ben Stride et al. 2024). Thus, the optimized protocol remains highly representative and effective for monitoring the most prevalent environmental MPs.

The recovery rate was also affected by the sediment matrix (Fig. 3). In detail, for all the tested polymers, the highest MPs recovery rate was achieved in medium and coarse sand matrices while a strong decrease was observed for finer sediments (fine sand and coarse silt). This pattern can be attributed to the physical and physicochemical properties of fine-grained sediments. In particular, finer particles tend to exhibit greater surface area to volume ratio and cohesiveness, which enhance the adsorption and entrapment of MPs within the sediment matrix. Additionally, the increased presence of clay and silt fractions promotes aggregation processes, thereby reducing the efficiency of density-based separation methods. As a result, MPs embedded within fine, cohesive sediments are less likely to detach and float during the extraction procedure, leading to lower apparent recovery rates. This highlights the importance of testing protocols on real, diverse matrices. Moreover, this aspect makes it difficult to compare our efficiency results to the data present in literature for other protocols as this aspect is generally neglected.

**Fig. 4:**
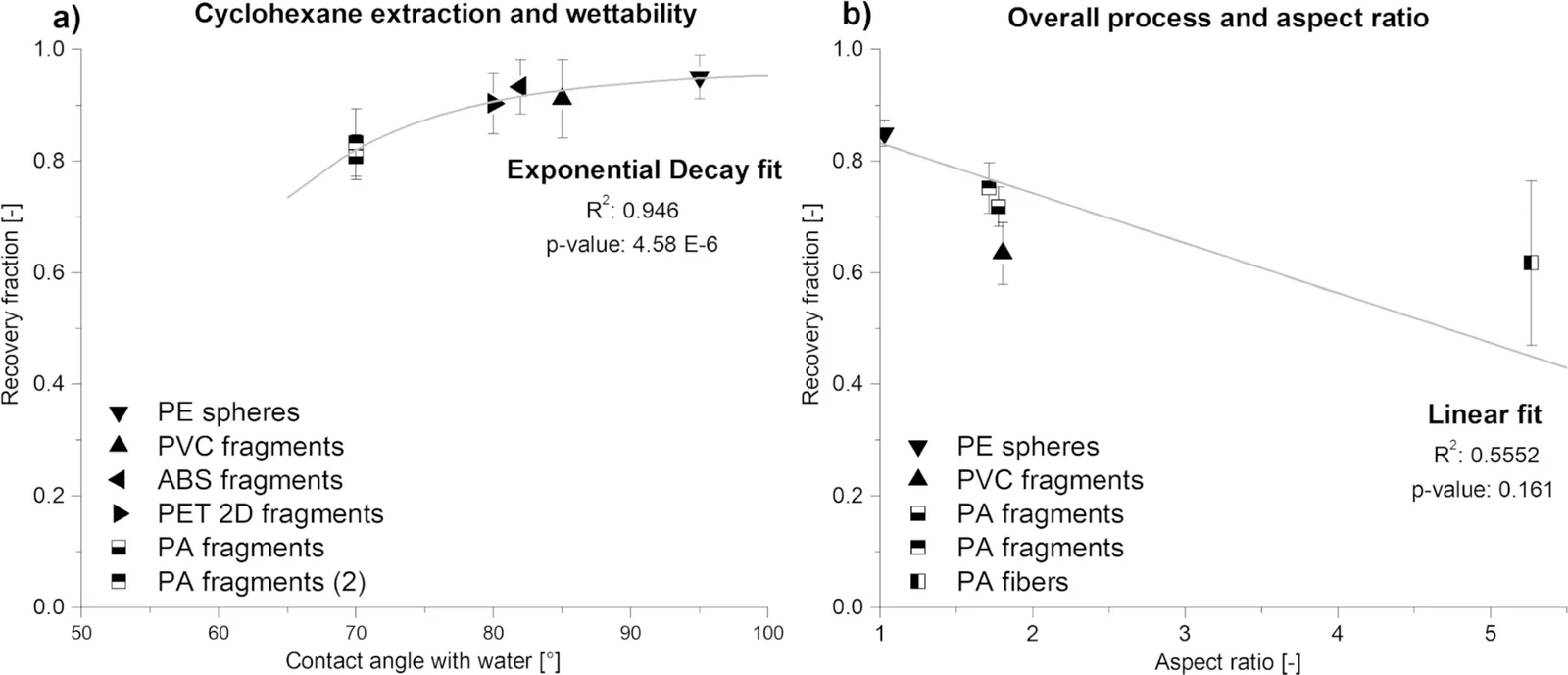
Influence of microplastic characteristics on recovery efficiency. **a** Cyclohexane extraction MP recovery fraction compared to polymer wettability (expressed as contact angle with water). **b** Overall MP recovery fraction compared with the AS of the MPs (Table S1) tested in fine sand. Exponential decay (**a**) and linear (**b**) fitting was presented (gray lines) together with the R^2^ and *p*-values. The tested polymers were: polyethylene (PE), polyvinyl chloride (PVC), acrylonitrile-butadiene styrene (ABS), polyamide (PA) and polyethylene terephthalate (PET). Numerical values are reported in Table S2

To isolate the efficiency of the proposed cyclohexane affinity extraction step respect to the polymers’ characteristics we evaluated its efficiency across different polymers (PE, PVC, PA and PET) with different wettability, size and AS (Table S1). An overall recovery efficiency of 0.89 ± 0.06 was observed with differences between the tested polymers (see Table S2 for details). To evaluate the relationship between the polymer surface characteristic and the recovery efficiency, we plotted the recovery fraction over the wettability of the polymers, expressed as the water contact angle (Fig. 4a). PA fibers were excluded from this evaluation due to their different shape (Table S1), which introduces other effects on recovery efficiency. A clear relationship between the recovery fraction and the wettability was found and mathematically modeled by an exponential decay fitting. The recovery is maximized for MPs with a water contact angle higher than 75–80°, while it decreases for more hydrophilic surfaces. This relationship highlights the efficiency of our method in a wide range of applications and moreover it is suggested the importance of testing MPs extraction protocol using polymers with different characteristics and properties.

To evaluate the effect of particle shape on our extraction process we plotted the overall recovery fraction against the AS of the MPs. In this case only MPs with a density both similar to each other and below that of the flotation solution used for the separation procedure (1.2 g/cm^3^) were investigated (i.e., PET and ABS were excluded) (Fig. 4b) in order also to neglect the limitations related to the NaCl solution density. No simple mathematic relationship was found between the MPs shape and the recovery, maybe due to other dimensional and morphological factors that complexly interact. However, a trend was observed suggesting that fibers are slightly harder to recover than the spheres or fragments.

Crucially, the lower recovery rate for PVC fragments (0.63 ± 0.06) in this specific test (Fig. 4b) should be interpreted as a “worst case scenario” validation. This result is primarily connected to the sediment characteristic used for this evaluation (i.e., fine sand) rather than a failure of the extraction itself. Indeed, the extraction efficiency of PVC fragments was found to be 0.86 in coarse sand and even higher (0.91) in clean matrices (Table S2). Testing the protocol under such adverse and complex matrix conditions underscores the reliability of the method for realistic environmental screening.

### Raman analysis and MPs identification

The unspiked aliquots of the sediment samples collected from the Arno River were processed using our extraction protocol to determine the environmental MPs concentration, thereby demonstrating the applicability of the method to real environmental samples.

**Fig. 5:**
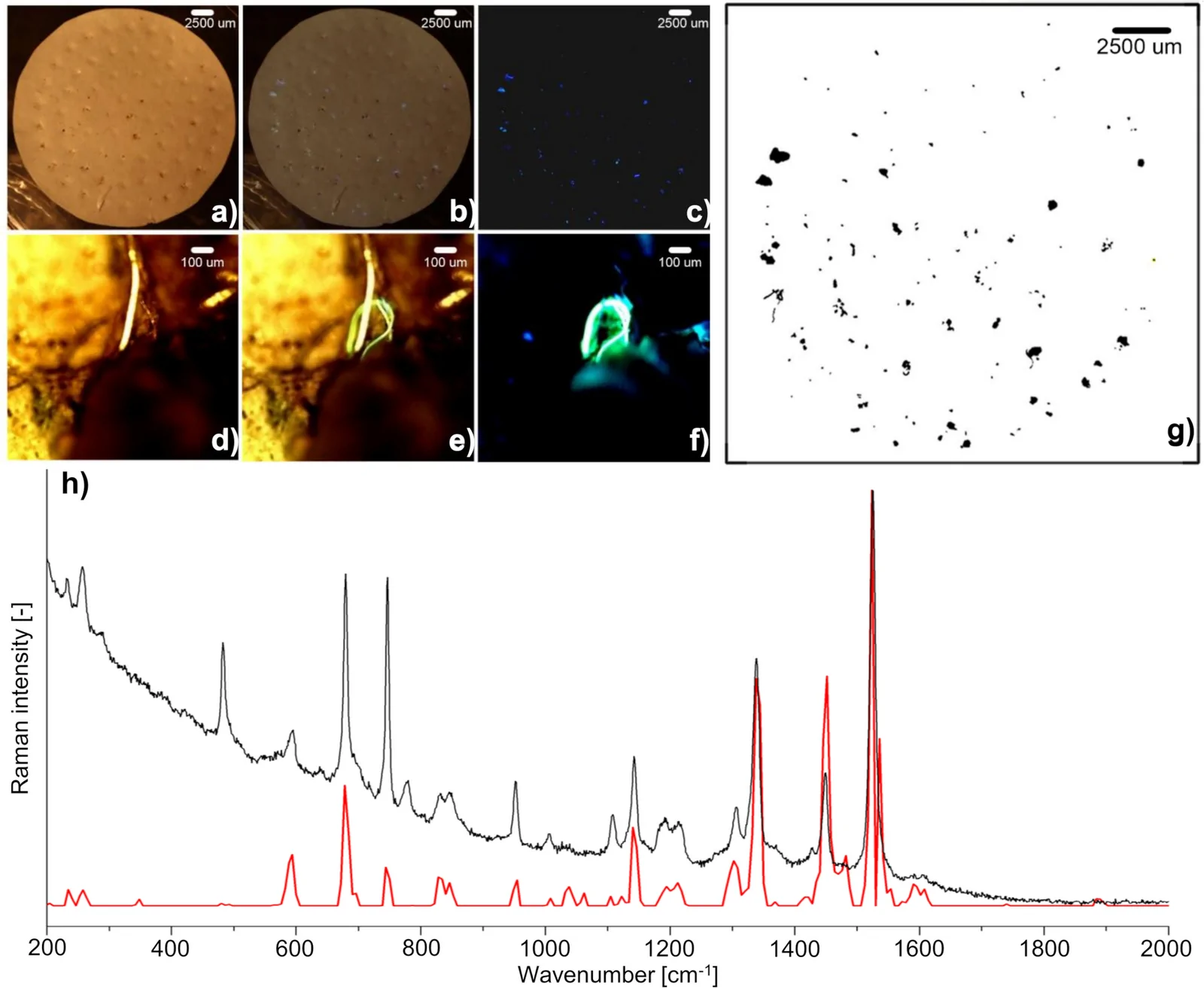
Image processing and Raman identification of microplastics. **a** RGB and **c** fluorescence (370-nm excitation) images of an analyzed filter, together with **b** their overlapped image and **g** the particle detection output of a filter processed using ImageJ software. In the figure, it is also represented the **d**–**f** RGB, fluorescence, and overlapped images of a selected polypropylene (PP) particle. **h** Raman spectrum (black line) of the identified PP particle compared with the OpenSpecy reference spectrum (red line) providing the best correlation coefficient

Initially, RGB and fluorescence (370 nm excitation) images of the filter holding the sample were acquired, combined and processed using ImageJ software to detect potential MPs (Fig. 5). This step sped up the procedure when dealing with reference materials in the screening of separation methods. Then, the filters were carefully examined under a microscope prior to acquiring the Raman spectra (see Chapter 2. *Materials and methods*). Notably, when the filters were analyzed using microRaman, no trace of cyclohexane or fluorescence interference that could affect the identification process was observed. An example of the Raman spectrum of environmental polypropylene MPs, found in a sample, is presented in Fig. 5. In the figure also the successfull match with the OpenSpecy reference library was shown.

**Fig. 6:**
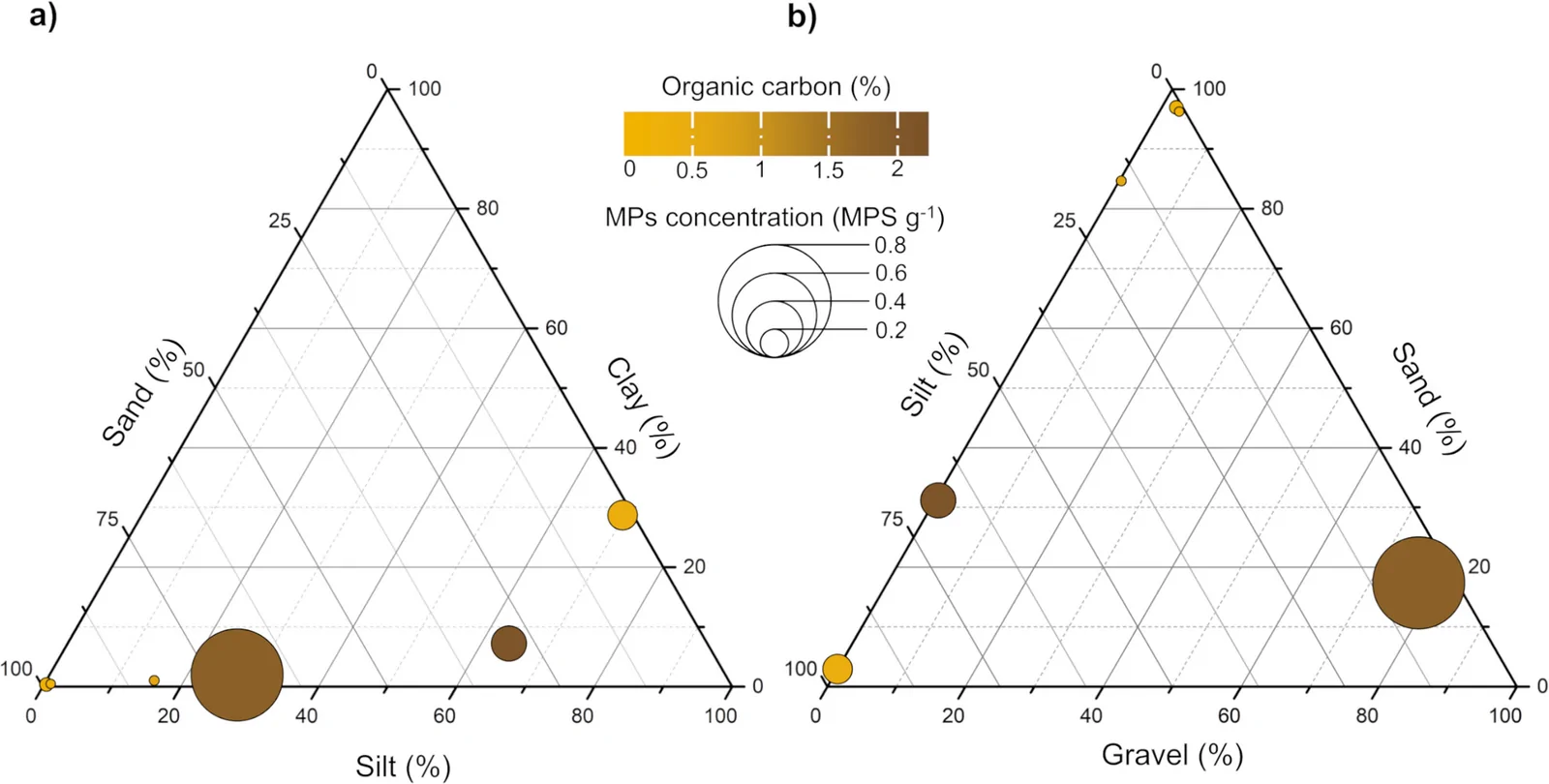
Environmental microplastic distribution in the Arno River. Distribution of the different samples according to their grain size distribution, organic carbon (% of dried weight sample) and microplastic occurrence expressed as numerical abundance (number of MP items per grams of dry sediment). The grain size distributions are reported as percentage of sand, clay and silt (**a**), or gravel, silt, and sand (**b**)

Overall, in the Arno River samples that we examined five different polymers were found: polypropylene (PP), polyester (PES), polyethylene terephthalate (PET), polycarbonate (PC), and polyethylene (PE) (Table S3). The MP concentration, expressed as numerical abundance (i.e., number of MPs per grams of dry sediment) varied widely ranging from 0.030 items g^−1^ observed in the fine sand sample to 0.653 items g^−1^ in the coarse gravel sample (Fig. 6 and Table S3). Across all samples, the median was 0.151 items g^−1^ and the mean was 0.217 ± 0.23 items g^−1^. The samples analyzed have different organic contents (from 0.15% in medium sand to 2.2% in medium silt) and different grain size distributions. In detail, the matrices ranged from primarily gravel composition (Coarse gravel, D_50_ 39.98 mm), to primarily sand composition (Coarse sand, D_50_ 0.610 mm and medium sand, D_50_ 0.460 mm and fine sand, D_50_ 0.247 mm) and primarily silt (Coarse silt, D_50_ 0.023 mm and fine/medium silt, D_50_ 0.015 mm). These findings successfully demonstrate the robustness of this extraction method across highly heterogeneous riverine sediment matrices with different grain size distribution and organic amount.

Although the primary goal of this study was the methodological optimization of the protocol, the preliminary data allow for a comparison with data previously reported in the literature. A direct comparison with Ghinassi et al. (2023), who investigated the MPs in the Arno Rivers alternate bars upstream Florence has been performed. In Ghinassi et al. (2023), MPs were extracted by H_2_O_2_ oxidation, density flotation and filtration. The MPs concentration, evaluated by the hot needle methods (Ruggero et al. 2020), was in the range of 0.44–5.68 items g^−1^, typical of highly polluted rivers, and significantly higher compared to our results. However, two major points must be evaluated to explain this discrepancy. First of all, the matrices investigated are different. The emerged sediment bars can capture and trap MPs transported during major flood events. Furthermore, also the inherent limitation of the hot needle methods must be evaluated. This method is well-known to overestimate the MPs concentration due to false positives (Beckingham et al. 2023; Gerigny et al. 2024). Our results are more consistent with studies of other, smaller, Tuscan rivers such as the Osa, Albegna and Ombrone rivers (0.045–1.069 items g^−1^) (Guerranti et al. 2017) or Mugnone river (0.5–1.54 items g^−1^) (Rimondi et al. 2022).

### Comparison with other sediment analysis techniques

To evaluate the performance of our extraction method, we compared it with previously reported protocols (Table 1). Our method has some issues connected with the low recovery efficiency of high-density polymers which is connected to the use of NaCl for the density separation (as previously discussed). However, it presents a significantly higher throughput and lower costs. As shown in Table 1, methods with higher throughput are limited to sediments with low organic content, such as marine sediments. In contrast, protocols for organic-rich sediments rely on complex enzymatic digestion methods or on oil-assisted extraction. However, these alternatives either compromise Raman identification or drastically increase costs and processing time. For example, the enzymatic digestion protocol provides high accuracy, but it requires much more time than our protocols.

**Table 1:** Comparison of different microplastic extraction protocols. Schematic representation of the proposed method and literature protocols for MPs extraction and identification. The comparison evaluates the matrices investigated, the method used to identify MPs, the recovery efficiencies (%), the dimensional range evaluated (in μm), throughput (i.e., the estimated processing time), the reagent price (€) and the environmental and health hazard of the chemical used. Information on how these data were obtained is reported in the SI. For sake of the reader, the corresponding flowcharts position in Fig. 2 are reported

Sample	Method	Technique used	Recovery efficiency (%)	Dimension evaluated (μm)	Throughput (estimated processing time)	Reagent price (€)	Environmental risk	Health hazard (toxicity)	Ref
Marine sediments	Density separation and filtration (Fig. 2a)	FT-IR	-	-	High (~1 day)	Negligible	No	No	Thompson et al. (2004)
Marine sediments	Green protocol (Fig. 2e)	Optical microscopy identification (stereomicroscopy, SEM, EDS, microRaman)	-	70–8000	High (< 1 h)	Negligible	No	No	Rossi et al. (2024)
Agricultural soils	Canola oil and unsaturated NaCl solution (Fig. 2c)	Incineration method	76–98	< 1000	High (< 1 day)	~15 € for 100 g of sediments	Low (H_2_O_2_)	Low (H_2_O_2_)	Kononov et al. (2022)
Soil, compost	Olive oil extraction (Fig. 2b)	FT-IR	89–95	5–1000	High (~3 days)	~30 € for 100 g of sediments	Moderate (H_2_O_2_, hexane)	Moderate (H_2_O_2_, hexane)	Scopetani et al. (2020)
Estuarine sediments	Enzymatic digestion (Fig. 2d)	FPA-mFT-IR	-	10–5000	Low (~24–28 days)	~357 € for 100 g of sediments	High (ZnCl_2_, H_2_O_2_)	High (ZnCl_2,_ H_2_O_2_)	Simon-Sánchez et al. (2024)
River sediments	Density separation and cyclohexane extraction (Fig. 2f)	microRaman spectroscopy	42–81	20–5000	Medium (~8 days)	~4,5 € for 100 g of sediments	Moderate (H_2_O_2_, cyclohexane)	Moderate (H_2_O_2_, cyclohexane)	This paper

Overall, our protocol is a compromise for large-scale monitoring where high amounts of sample have to be investigated. This approach is consistent with the growing European regulatory interest in harmonized basin-scale monitoring of microplastics within the framework of the Water Framework Directive (European Commission 2022; Galgani et al. 2024).

## Conclusions

Full evaluation of the MP pollution strongly depends on having robust and precise data on its presence in environmental samples. Systematic, large-scale monitoring of riverine sediments is particularly important for this reason. However, the nature of the riverine sediment matrices, cohesive and with a high organic matter content, complicates the analysis and requires adequate approaches. Previous protocols are possibly either too slow/expensive or not adequate for large-scale applications on these matrices.

Here, we optimized a highly cost-effective and fast method for the investigation of MPs in river sediments by Raman spectroscopy. This offers an optimal trade-off between processing efficiency, time, and cost. We combined Fenton oxidation with a cyclohexane affinity extraction to obtain a reduction of processing time and reagent costs essential for application to basin-scale studies. This paper also represents a step for the harmonization of MP_S_ extraction methods. Attention was dedicated to the development of a method able to maximize the effectiveness of Raman spectroscopy methods by limitation of the fluorescence background sources. As further development, other solvents should be tested in order to improve effectiveness and eco-compatibility in the MPs solvent extraction step while keeping the Raman fluorescence background as low as possible. Furthermore, we suggest that a different salt for density separation could expand the applicability of the method to target, if necessary, specific high-density polymer MPs.

## Supplementary Information

Below is the link to the electronic supplementary material.

ESM 1 (PDF 0.98 MB)

## Data Availability

Data and details on the custom-made laboratory glassware will be made available upon request.

## Abbreviations

*AS*: Aspect ratio
*MP*: Microplastic
*ABS*: Acrylonitrile-butadiene styrene
*FT-IR*: Fourier-transform infrared
*MAPs*: Macroplastics
*MEPs*: Mesoplastics
*NR*: Nile red
*PA*: Polyamide
*PC*: Polycarbonate
*PE*: Polyethylene
*PET*: Polyethylene terephthalate
*PES*: Polyester
*PP*: Polypropylene
*POPs*: Persistent organic pollutants
*PVC*: Polyvinyl chloride
*RS*: Raman spectroscopy

## References

[CR1] Alder CM, Hayler JD, Henderson RK, Redman AM, Shukla L, Shuster LE, Sneddon HF (2016) Updating and further expanding GSK’s solvent sustainability guide. Green Chem 18(13):3879–3890. 10.1039/C6GC00611F

[CR2] Andrady AL (2022) Weathering and fragmentation of plastic debris in the ocean environment. Mar Pollut Bull 180:113761. 10.1016/j.marpolbul.2022.113761 35665618

[CR3] Beckingham B, Apintiloaiei A, Moore C, Brandes J (2023) Hot or not: systematic review and laboratory evaluation of the hot needle test for microplastic identification. Microplastics Nanoplastics 3(1):8. 10.1186/s43591-023-00056-4

[CR4] Becucci M, Mancini M, Campo R, Paris E (2022) Microplastics in the Florence wastewater treatment plant studied by a continuous sampling method and Raman spectroscopy: a preliminary investigation. Sci Total Environ 808:152025. 10.1016/j.scitotenv.2021.152025 34856255

[CR5] Brander SM, Renick VC, Foley MM, Steele C, Woo M, Lusher A, Carr S, Helm P, Box C, Cherniak S, Andrews RC, Rochman CM (2020) Sampling and quality assurance and quality control: a guide for scientists investigating the occurrence of microplastics across matrices. Appl Spectrosc 74(9):1099–1125. 10.1177/0003702820945713 32643389

[CR6] Cantasano N (2022) Marine pollution by microplastics in the Mediterranean Sea. J Mar Sci Eng 10(7):858. 10.3390/jmse10070858

[CR7] Casadio F, Leona M, Lombardi JR, Van Duyne R (2010) Identification of organic colorants in fibers, paints, and glazes by surface enhanced Raman spectroscopy. Acc Chem Res 43(6):782–791. 10.1021/ar100019q 20420359

[CR8] Chakraborty I, Banik S, Biswas R, Yamamoto T, Noothalapati H, Mazumder N (2023) Raman spectroscopy for microplastic detection in water sources: a systematic review. Int J Environ Sci Technol 20(9):10435–10448. 10.1007/s13762-022-04505-0

[CR9] Chang M, Wen Q, Bai T, Liu Y, Huang X, Dai Z, Sun S, Yang Y, Li C (2023) Novel efficient method to continuously collect microplastics from seawater via a two-phase system. Environ Sci Technol Lett 10(10):924–930. 10.1021/acs.estlett.3c00546

[CR10] Chatterjee S, Krolis E, Molenaar R, Claessens MMAE, Blum C (2023) Nile red staining for nanoplastic quantification: overcoming the challenge of false positive counts due to fluorescent aggregates. Environ Chall 13:100744. 10.1016/j.envc.2023.100744

[CR11] Cole M, Lindeque P, Fileman E, Halsband C, Goodhead R, Moger J, Galloway TS (2013) Microplastic ingestion by zooplankton. Environ Sci Technol 47(12):6646–6655. 10.1021/es400663f 23692270

[CR12] Cole M, Coppock R, Lindeque PK, Altin D, Reed S, Pond DW, Sørensen L, Galloway TS, Booth AM (2019) Effects of nylon microplastic on feeding, lipid accumulation, and moulting in a coldwater copepod. Environ Sci Technol 53(12):7075–7082. 10.1021/acs.est.9b01853 PMC7007202 31125216

[CR13] Cottom JW, Cook E, Velis CA (2024) A local-to-global emissions inventory of macroplastic pollution. Nature 633(8028):101–108. 10.1038/s41586-024-07758-6 PMC11374682 39232151

[CR14] Covernton GA, Davies HL, Cox KD, El-Sabaawi R, Juanes F, Dudas SE, Dower JF (2021) A Bayesian analysis of the factors determining microplastics ingestion in fishes. J Hazard Mater 413:125405. 10.1016/j.jhazmat.2021.125405 33930957

[CR15] Cowger W, Karapetrova A, Lincoln C, Chamas A, Sherrod H, Leong N, Lasdin KS, Knauss C, Teofilović V, Arienzo MM, Steinmetz Z, Primpke S, Darjany L, Murphy-Hagan C, Moore S, Moore C, Lattin G, Gray A, Kozloski R, Bryksa J, Maurer B (2025) Open specy 1.0: automated (hyper)spectroscopy for microplastics. Anal Chem 97(32):17345–17356. 10.1021/acs.analchem.5c00962 40788199

[CR16] D’Avignon G, Gregory-Eaves I, Ricciardi A (2022) Microplastics in lakes and rivers: an issue of emerging significance to limnology. Environ Rev 30(2):228–244. 10.1139/er-2021-0048

[CR17] Dalvand M, Hamidian AH (2023) Occurrence and distribution of microplastics in wetlands. Sci Total Environ 862:160740. 10.1016/j.scitotenv.2022.160740 36496018

[CR18] Dikareva N, Simon KS (2024) Factors controlling transport dynamics of microplastics in streams. ACS EST Water 4(9):4120–4128. 10.1021/acsestwater.4c00393

[CR19] Ding T, Wei L, Hou Z, Li J, Zhang C, Lin D (2022) Microplastics altered contaminant behavior and toxicity in natural waters. J Hazard Mater 425:127908. 10.1016/j.jhazmat.2021.127908 34883377

[CR20] Dris R, Imhof H, Sanchez W, Gasperi J, Galgani F, Tassin B, Laforsch C (2015) Beyond the ocean: contamination of freshwater ecosystems with (micro-)plastic particles. Environ Chem 12(5):539–550. 10.1071/EN14172

[CR21] Dris R, Gasperi J, Rocher V, Tassin B (2018) Synthetic and non-synthetic anthropogenic fibers in a river under the impact of Paris Megacity: sampling methodological aspects and flux estimations. Sci Total Environ 618:157–164. 10.1016/j.scitotenv.2017.11.009 29128764

[CR22] Elkhatib D, Oyanedel-Craver V (2020) A critical review of extraction and identification methods of microplastics in wastewater and drinking water. Environ Sci Technol 54(12):7037–7049. 10.1021/acs.est.9b06672 32432459

[CR23] Ellison AH, Zisman WA (1954) Wettability studies on nylon, polyethylene terephthalate and polystyrene. J Phys Chem 58(6):503–506. 10.1021/j150516a013

[CR24] Emberson-Marl H, Coppock RL, Cole M, Godley BJ, Mimpriss N, Nelms SE, Lindeque PK (2023) Microplastics in the Arctic: a transect through the Barents Sea. Front Mar Sci. 10.3389/fmars.2023.1241829

[CR25] European Chemicals Agency (ECHA) (2019) Annex XV restriction report: proposal for a restriction – intentionally added microplastics. Version 1, 11 January 2019. European Chemicals Agency, Helsinki, Finland.

[CR26] European Commission (2022) Proposal for a directive of the european parliament and of the council amending directive 2000/60/EC establishing a framework for community action in the field of water policy, directive 2006/118/EC on the protection of groundwater against pollution and deterioration and directive 2008/105/EC on environmental quality standards in the field of water policy. COM(2022) 540 final, 2022/0344(COD), Brussels, Belgium

[CR27] Felsing S, Kochleus C, Buchinger S, Brennholt N, Stock F, Reifferscheid G (2018) A new approach in separating microplastics from environmental samples based on their electrostatic behavior. Environ Pollut 234:20–28. 10.1016/j.envpol.2017.11.013 29154206

[CR28] Galgani F, Lusher AL, Strand J, Haarr ML, Vinci M, Molina Jack E, Kagi R, Aliani S, Herzke D, Nikiforov V, Primpke S, Schmidt N, Fabres J, De Witte B, Solbakken VS, van Bavel B (2024) Revisiting the strategy for marine litter monitoring within the european marine strategy framework directive (MSFD). Ocean Coast Manag 255:107254. 10.1016/j.ocecoaman.2024.107254

[CR29] Gardon T, Reisser C, Soyez C, Quillien V, Le Moullac G (2018) Microplastics affect energy balance and gametogenesis in the pearl oyster *Pinctada margaritifera*. Environ Sci Technol 52(9):5277–5286. 10.1021/acs.est.8b00168 29620881

[CR30] Gerigny O, Blanco G, Lips U, Buhhalko N, Chouteau L, Georges E, Meyers N, Vanavermaete D, Galgani F, Ourgaud M, Papillon L, Sempéré R, De Witte B (2024) Comparative analysis of microplastics detection methods applied to marine sediments: a case study in the Bay of Marseille. Mar Pollut Bull 207:116787. 10.1016/j.marpolbul.2024.116787 39146714

[CR31] Geyer R, Jambeck JR, Law KL (2017) Production, use, and fate of all plastics ever made. Sci Adv. 10.1126/sciadv.1700782 PMC5517107 28776036

[CR32] Ghinassi M, Michielotto A, Uguagliati F, Zattin M (2023) Mechanisms of microplastics trapping in river sediments: insights from the Arno river (Tuscany, Italy). Sci Total Environ 866:161273. 10.1016/j.scitotenv.2022.161273 36610632

[CR33] Grbic J, Nguyen B, Guo E, You JB, Sinton D, Rochman CM (2019) Magnetic extraction of microplastics from environmental samples. Environ Sci Technol Lett 6(2):68–72. 10.1021/acs.estlett.8b00671

[CR34] Guerranti C, Cannas S, Scopetani C, Fastelli P, Cincinelli A, Renzi M (2017) Plastic litter in aquatic environments of Maremma Regional Park (Tyrrhenian Sea, Italy): contribution by the Ombrone river and levels in marine sediments. Mar Pollut Bull 117(1–2):366–370. 10.1016/j.marpolbul.2017.02.021 28202278

[CR35] Hermsen E, Mintenig SM, Besseling E, Koelmans AA (2018) Quality criteria for the analysis of microplastic in biota samples: a critical review. Environ Sci Technol 52(18):10230–10240. 10.1021/acs.est.8b01611 PMC6146318 30137965

[CR36] Hidalgo-Ruz V, Gutow L, Thompson RC, Thiel M (2012) Microplastics in the marine environment: a review of the methods used for identification and quantification. Environ Sci Technol 46(6):3060–3075. 10.1021/es2031505 22321064

[CR37] Huang M, Si C, Qiu C, Wang G (2024) Microplastics analysis: from qualitative to quantitative. Environ Sci Adv 3(12):1652–1668. 10.1039/D4VA00244J

[CR38] Idehara W, Haga Y, Tsujino H, Ikuno Y, Manabe S, Hokaku M, Asahara H, Higashisaka K, Tsutsumi Y (2025) Exploring Nile Red staining as an analytical tool for surface-oxidized microplastics. Environ Res 269:120934. 10.1016/j.envres.2025.120934 39862951

[CR39] Jambeck J, Geyer R, Wilcox C, Siegler TR, Perryman M, Andrady A, Narayan R, Lavender Law K (2015) Plastic waste inputs from land into the ocean. Science 347(6223):768–771. 10.1126/science.1260352 25678662

[CR40] Jefroy M, Lusher A, van Bavel B, Ghaffari P (2026) Microplastics in sediments: a systematic review structured through reproducible analytical pipelines. Chemosphere 396:144849. 10.1016/j.chemosphere.2026.144849 41619516

[CR41] Jewett E, Arnott G, Connolly L, Vasudevan N, Kevei E (2022) Microplastics and their impact on reproduction—can we learn from the *C. elegans* model? Front Toxicol. 10.3389/ftox.2022.748912 PMC8987311 35399297

[CR42] Jiao M, Cao S, Ren L, Li R (2021) Analysis of composite microplastics in sediment using 3D Raman spectroscopy and imaging method. J Hazard Mater Adv 3:100016. 10.1016/j.hazadv.2021.100016

[CR43] Julienne F, Delorme N, Lagarde F (2019) From macroplastics to microplastics: role of water in the fragmentation of polyethylene. Chemosphere 236:124409. 10.1016/j.chemosphere.2019.124409 31545205

[CR44] Kedzierski M, Le Tilly V, César G, Sire O, Bruzaud S (2017) Efficient microplastics extraction from sand. A cost effective methodology based on sodium iodide recycling. Mar Pollut Bull 115(1–2):120–129. 10.1016/J.MARPOLBUL.2016.12.002 28040250

[CR45] Kononov A, Hishida M, Suzuki K, Harada N (2022) Microplastic extraction from agricultural soils using canola oil and unsaturated sodium chloride solution and evaluation by incineration method. Soil Syst 6(2):54. 10.3390/soilsystems6020054

[CR46] Kooi M, Koelmans AA (2019) Simplifying microplastic via continuous probability distributions for size, shape, and density. Environ Sci Technol Lett 6(9):551–557. 10.1021/acs.estlett.9b00379

[CR47] Kooi M, Primpke S, Mintenig SM, Lorenz C, Gerdts G, Koelmans AA (2021) Characterizing the multidimensionality of microplastics across environmental compartments. Water Res 202:117429. 10.1016/j.watres.2021.117429 34304075

[CR48] Kotula AP, Orski SV, Brignac KC, Lynch JM, Heilala BMJ (2022) Time-gated Raman spectroscopy of recovered plastics. Mar Pollut Bull 181:113894. 10.1016/j.marpolbul.2022.113894 35785722

[CR49] Kumar A, Madan M, Kumar V, Bhattacharya J, Dubey KK (2026) Challenges and prospects in the identification of micro and nano plastics using Raman spectroscopy: a comprehensive review. Front Water. 10.3389/frwa.2026.1763916

[CR50] Lebreton L, Kooi M, Mani T, Mintenig SM, Tekman MB, van Emmerik T, Wolter H (2022) Plastics and the ocean : origin, characterization, fate, and impacts. John Wiley & Sons, Inc.

[CR51] Li Y, Fu J, Peng L, Sun X, Wang G, Wang Y, Chen L (2023) A sustainable emulsion for separation and Raman identification of microplastics and nanoplastics. Chem Eng J 469:143992. 10.1016/j.cej.2023.143992

[CR52] Lin C-H, Chang Y-F (2022) Comparison and characterization of pigments and dyes by Raman spectroscopy. Anal Sci 38(3):483–495. 10.2116/analsci.21SAR03 35359266

[CR53] Liu F, Vianello A, Vollertsen J (2019) Retention of microplastics in sediments of urban and highway stormwater retention ponds. Environ Pollut 255:113335. 10.1016/j.envpol.2019.113335 31604201

[CR54] Liu J, Zhang X, Du Z, Luan Z, Li L, Xi S, Wang B, Cao L, Yan J (2020) Application of confocal laser Raman spectroscopy on marine sediment microplastics. J Oceanol Limnol 38(5):1502–1516. 10.1007/s00343-020-0129-z

[CR55] Liu Y, Jiang W-Y, Liao Y, Sun R, Hu J, Lu Z, Chang M, Yang J, Dai Z, Zhou C, Hong P, Qian Z-J, Sun S, Ren L, Liang Y-Q, Zhang Y, Li C (2022) Separation of false-positive microplastics and analysis of microplastics via a two-phase system combined with confocal Raman spectroscopy. J Hazard Mater 440:129803. 10.1016/j.jhazmat.2022.129803 36027743

[CR56] Liu Y, Lin L, Yang B, Huang M, Huang X, Chen X, Dai Z, Sun S, Yang Y, Li C (2024) Separation and identification of nanoplastics via a two-phase system combined with surface-enhanced Raman spectroscopy. ACS Sustain Chem Eng 12(4):1595–1604. 10.1021/acssuschemeng.3c06891

[CR57] Mancini M, Soler M, Colomer J, Solari L, Serra T (2025) Synthetic microfibers driven by turbidity currents: transition from smooth bed to macro-roughness. Mar Pollut Bull 221:118509. 10.1016/j.marpolbul.2025.118509 40738039

[CR58] Miller ME, Kroon FJ, Motti CA (2017) Recovering microplastics from marine samples: a review of current practices. Mar Pollut Bull 123(1–2):6–18. 10.1016/j.marpolbul.2017.08.058 28886920

[CR59] Monnanni A, Rimondi V, Morelli G, Nannoni A, Cincinelli A, Martellini T, Chelazzi D, Laurati M, Sforzi L, Ciani F, Lattanzi P, Costagliola P (2024) Microplastics and microfibers contamination in the Arno River (Central Italy): impact from urban areas and contribution to the Mediterranean Sea. Sci Total Environ 955:177113. 10.1016/j.scitotenv.2024.177113 39490839

[CR60] Naiman RJ, Décamps H (1997) The ecology of interfaces: riparian zones. Annu Rev Ecol Syst 28(1):621–658. 10.1146/annurev.ecolsys.28.1.621

[CR61] Nava V, Leoni B (2021) A critical review of interactions between microplastics, microalgae and aquatic ecosystem function. Water Res 188:116476. 10.1016/j.watres.2020.116476 33038716

[CR62] Nikhil VG, Amritha GG, Ranjeet K, Varghese GK (2024) Distribution of microplastics in seafloor sediments and their differential assimilation in nearshore benthic molluscs along the south-west coast of India. Environ Pollut 344:123350. 10.1016/j.envpol.2024.123350 38219899

[CR63] Okoye CO, Addey CI, Oderinde O, Okoro JO, Uwamungu JY, Ikechukwu CK, Okeke ES, Ejeromedoghene O, Odii EC (2022) Toxic chemicals and persistent organic pollutants associated with micro-and nanoplastics pollution. Chem Eng J Adv 11:100310. 10.1016/j.ceja.2022.100310

[CR64] Peruzzi C, Castaldi M, Francalanci S, Solari L (2019) Three‐dimensional hydraulic characterisation of the Arno River in Florence. J Flood Risk Manag. 10.1111/jfr3.12490

[CR65] Pita F, Castilho A (2020) Separation of PET from other plastics by flotation combined with alkaline pretreatment. Polímeros 30(3):e2020026. 10.1590/0104-1428.03320

[CR66] Plastics Europe (2022) Plastics – the facts 2022: an analysis of european plastics production, demand, conversion and end-of-life management. Plastics Europe, Brussels, Belgium

[CR67] Prasad S, Bennett A, Triantafyllou M (2024) Characterization of Nile Red-stained microplastics through fluorescence spectroscopy. J Mar Sci Eng 12(8):1403. 10.3390/jmse12081403

[CR68] Prat D, Wells A, Hayler J, Sneddon H, McElroy CR, Abou-Shehada S, Dunn PJ (2016) CHEM21 selection guide of classical- and less classical-solvents. Green Chem 18(1):288–296. 10.1039/C5GC01008J

[CR69] Qin Y, Song F, Ai Z, Zhang P, Zhang L (2015) Protocatechuic acid promoted alachlor degradation in Fe(III)/H_2_O_2_ fenton system. Environ Sci Technol 49(13):7948–7956. 10.1021/es506110w 26066010

[CR70] Rambacher J, Pantos O, Hardwick S, Cameron EZ, Gaw S (2023) Transforming encounters: a review of the drivers and mechanisms of macrofaunal plastic fragmentation in the environment. Camb Prisms Plast 1:1–33. 10.1017/plc.2023.6

[CR71] Rimondi V, Monnanni A, De Beni E, Bicocchi G, Chelazzi D, Cincinelli A, Fratini S, Martellini T, Morelli G, Venturi S, Lattanzi P, Costagliola P (2022) Occurrence and quantification of natural and microplastic items in urban streams: the case of Mugnone Creek (Florence, Italy). Toxics 10(4):159. 10.3390/toxics10040159 PMC9025813 35448420

[CR72] Rodrigues MO, Gonçalves AMM, Gonçalves FJM, Abrantes N (2020) Improving cost-efficiency for MPs density separation by zinc chloride reuse. MethodsX 7:100785. 10.1016/j.mex.2020.100785 PMC6997619 32025505

[CR73] Rohais S, Armitage JJ, Romero-Sarmiento M-F, Pierson J-L, Teles V, Bauer D, Cassar C, Sebag D, Klopffer M-H, Pelerin M (2024) A source-to-sink perspective of an anthropogenic marker: a first assessment of microplastics concentration, pathways, and accumulation across the environment. Earth Sci Rev 254:104822. 10.1016/j.earscirev.2024.104822

[CR74] Rossi M, Vergara A, Capozzi F, Giordano S, Spagnuolo V, Troisi R, Vedi V, Ambrosi de Magistris F, Fiaschini N, Tommasi T, Guida M, D’Aniello M, Donadio C (2024) A new green protocol for the identification of microplastics and microfibers in marine sediments, a case study from the Vesuvian Coast, Southern Italy. J Hazard Mater 477:135272. 10.1016/j.jhazmat.2024.135272 39098199

[CR75] Ruggero F, Gori R, Lubello C (2020) Methodologies for microplastics recovery and identification in heterogeneous solid matrices: a review. J Polym Environ 28(3):739–748. 10.1007/s10924-019-01644-3

[CR76] Sancataldo G, Avellone G, Vetri V (2020) Nile River red lifetime reveals microplastic identity. Environ Sci Process Impacts 22(11):2266–2275. 10.1039/D0EM00348D 33064112

[CR77] Schmidt C, Krauth T, Wagner S (2017a) Correction to export of plastic debris by rivers into the sea. Environ Sci Technol 51(21):12246–12253. 10.1021/acs.est.7b02368 29019247

[CR78] Schmidt C, Krauth T, Wagner S (2017b) Export of plastic debris by rivers into the sea. Environ Sci Technol 51(21):12246–12253. 10.1021/acs.est.7b02368 29019247

[CR79] Scopetani C, Chelazzi D, Mikola J, Leiniö V, Heikkinen R, Cincinelli A, Pellinen J (2020) Olive oil-based method for the extraction, quantification and identification of microplastics in soil and compost samples. Sci Total Environ 733:139338. 10.1016/j.scitotenv.2020.139338 32446078

[CR80] Simon-Sánchez L, Vianello A, Kirstein IV, Molazadeh M-S, Lorenz C, Vollertsen J (2024) Assessment of microplastic pollution and polymer risk in the sediment compartment of the Limfjord, Denmark. Sci Total Environ 950:175017. 10.1016/j.scitotenv.2024.175017 39059658

[CR81] Stanton T, Johnson M, Nathanail P, Gomes RL, Needham T, Burson A (2019) Exploring the efficacy of Nile Red in microplastic quantification: a costaining approach. Environ Sci Technol Lett 6(10):606–611. 10.1021/acs.estlett.9b00499

[CR82] Stride B, Abolfathi S, Bending GD, Pearson J (2024) Quantifying microplastic dispersion due to density effects. J Hazard Mater 466:133440. 10.1016/j.jhazmat.2024.133440 38246058

[CR83] Thompson RC, Olsen Y, Mitchell RP, Davis A, Rowland SJ, John AWG, McGonigle D, Russell AE (2004) Lost at sea: where is all the plastic? Science (1979) 304(5672):838–838. 10.1126/science.1094559 15131299

[CR84] Thompson RC, Swan SH, Moore CJ, vom Saal FS (2009) Our plastic age. Philos Trans R Soc B Biol Sci 364(1526):1973–1976. 10.1098/rstb.2009.0054 PMC2874019 19528049

[CR85] Tumwesigye E, Felicitas Nnadozie C, C Akamagwuna F, Siwe Noundou X, William Nyakairu G, Odume ON (2023) Microplastics as vectors of chemical contaminants and biological agents in freshwater ecosystems: current knowledge status and future perspectives. Environ Pollut 330:121829. 10.1016/j.envpol.2023.121829 37196837

[CR86] Vannote RL, Minshall GW, Cummins KW, Sedell JR, Cushing CE (1980) The river continuum concept. Can J Fish Aquat Sci 37(1):130–137. 10.1139/f80-017

[CR87] Vianello A, Da Ros L, Boldrin A, Marceta T, Moschino V (2018) First evaluation of floating microplastics in the Northwestern Adriatic Sea. Environ Sci Pollut Res 25(28):28546–28561. 10.1007/s11356-018-2812-6 30091074

[CR88] Villa B, Bolla G, Gambino I, Terzaghi E, Boldrocchi G, Baldini E, Brusa F, Federici S, Ducoli S, Bergna G, Zoccali A, Malpei F, Di Guardo A, Bettinetti R (2025) Co-staining microplastics with Nile Red and Rose Bengal for improved optical quantification. Sci Rep 16(1):2917. 10.1038/s41598-025-32829-7 PMC12830938 41436592

[CR89] Weber CJ, Lechthaler S (2021) Plastics as a stratigraphic marker in fluvial deposits. Anthropocene 36:100314. 10.1016/j.ancene.2021.100314

[CR90] Witzig CS, Földi C, Wörle K, Habermehl P, Pittroff M, Müller YK, Lauschke T, Fiener P, Dierkes G, Freier KP, Zumbülte N (2020) When good intentions go bad—false positive microplastic detection caused by disposable gloves. Environ Sci Technol 54(19):12164–12172. 10.1021/acs.est.0c03742 32882126

[CR91] Xu J, Zuo R, Wu G, Liu J, Liu J, Huang C, Wang Z (2024) Global distribution, drivers, and potential hazards of microplastics in groundwater: a review. Sci Total Environ 954:176194. 10.1016/j.scitotenv.2024.176194 39270874

[CR92] Xue Q, Yu G, Lu F, Dong Y (2024) Fluorescent labelling combined with confocal differential Raman spectroscopy to detect microplastics in seawater. Spectrochim Acta A Mol Biomol Spectrosc 320:124591. 10.1016/j.saa.2024.124591 38850818

