# Supplementary material for: Advances in the sample pretreatment for determination of microplastics in riverbed sediments by Raman spectroscopy
Source: Environ Sci Pollut Res Int. 2026 Sep 25;33(31):16012–28. doi: 10.1007/s11356-026-38231-8 (PMC13642412; doi:10.1007/s11356-026-38231-8)
Supplement: Supplementary file 1 [file 11356_2026_38231_MOESM1_ESM.pdf]

Supplementary material

Advances in the sample pretreatment for determination of microplastics in riverbed sediments by Raman spectroscopy

Andrea Dali[1]*, Mirco Mancini[2]*, Daniela Santianni[3], Luca Solari[2] and Maurizio Becucci[1,4#]

[1]*Department of Chemistry "Ugo Schiff" (DICUS), University of Florence, Via della Lastruccia 3-13, I-50019 Sesto Fiorentino (FI), Italy*

[2]*Department of Civil and Environmental Engineering, University of Florence, Via Santa Marta 3, I-50139 Florence, Italy*

[3]*Publiacqua S.P.A., Via Villamagna 90C, I-50126 Florence, Italy*

[4]*INSTM, Research Unit of Florence, Via della Lastruccia 3, I-50019 Sesto Fiorentino (FI), Italy*

*Authors indicate with * equally contributed.*

[#]*Correspondence to: Maurizio Becucci: maurizio.becucci@unifi.it.*

Note to Table 1

Recovery efficiency and **Dimension evaluated:** when they are not indicated in the paper a dash was reported. The range reported evaluated the minimum and maximum dimensions of MPs investigated in the studies. If not clearly stated in the paper, they are considered as the minimum dimension considered and the larger mesh size sieve used in the studies. **Reagent price:** they are estimated based on quotes from Sigma-Aldrich for Italy (dated April 2026). Please note that prices may vary across different countries and suppliers and it depends also on reagent purity. Cost estimates refer strictly to the quantities of reagents described in the respective protocols; where not explicitly stated, the mass/volume of regents used was assumed based on the beaker used for the experiments or the amount of samples investigated. These estimates exclude the costs of water (e.g., Milli-Q), filters, analytical instrumentation, laboratory consumables, and sampling efforts considering the reagent's price. It is neglected the possibility to reuse solvent in further analysis. For comparability, prices are reported per 100 g of sample. **Throughput (estimated processing time):** it represents the estimated time, based on the protocol described, to make the sample ready for the analysis. The time necessary to collect the sample, drying it and to investigate it are not considered. **Environmental risk** and **health hazard (toxicity):** these levels are qualitatively assessed based on the Safety Data Sheets (SDS classification) of the reagents used which are indicated between brackets. H_2O_2 is a strong oxidizer and corrosive (ranging from Category 4 to Category 1 for the health hazard) and its health hazard is considered low due to the possibility of using protection system and careful handling. Regarding its environmental risk it is considered in category 3 and therefore, a low risk is reported. Cyclohexane and hexane are in category 1 for health and environmental risk, but a low amount is employed and therefore a moderate risk is considered. $ZnCl_2$ is in category 1 for health and environmental risk, and a high volume is employed and therefore a high risk is considered.

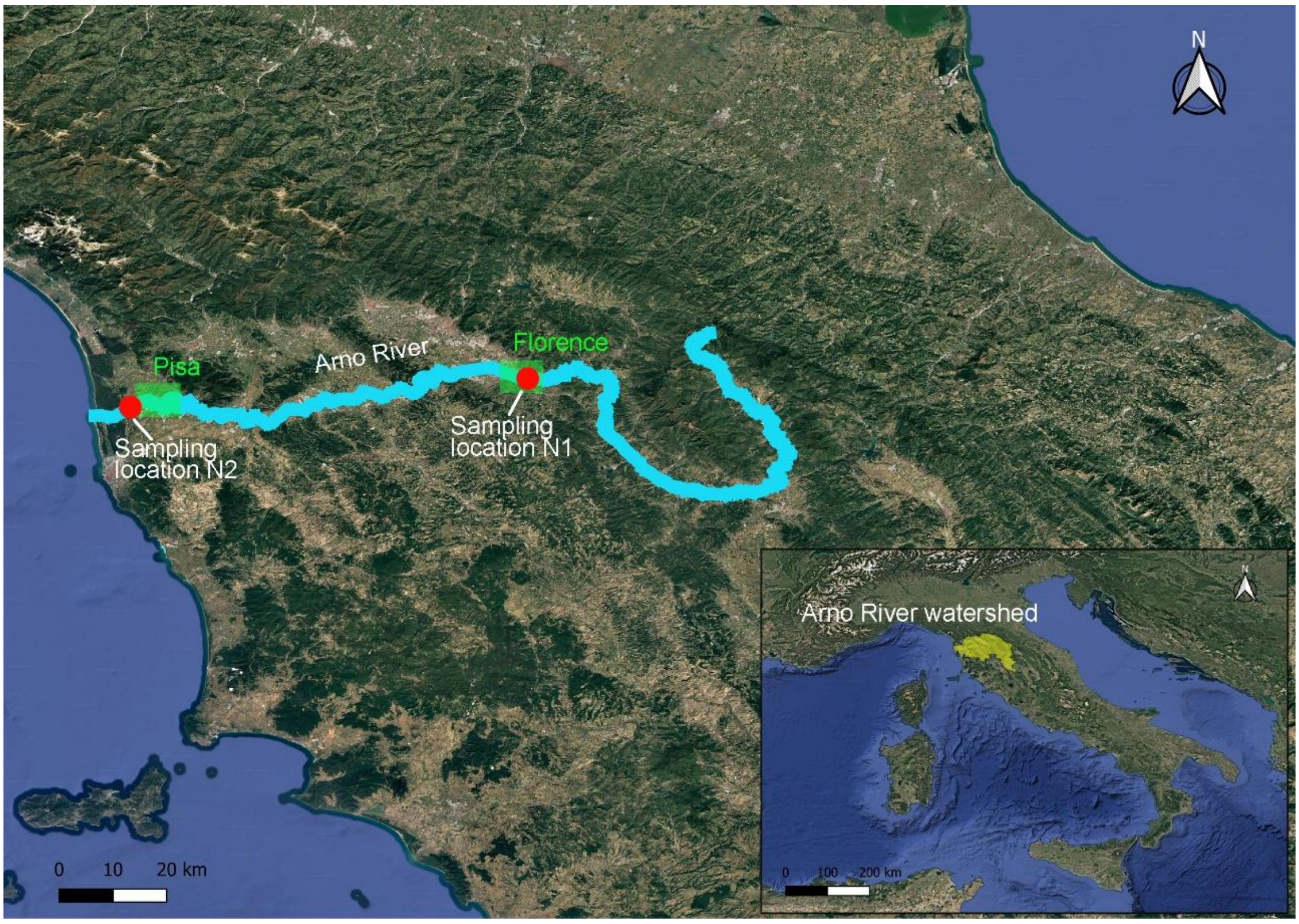

Fig. S1 Sampling locations along the Arno River. Map of Italy highlighting the Arno River (light blue), its watershed (yellow) and sampling sites (in red), distributed along the river stretch flowing through the cities of Florence (sampling location N1) and Pisa (sampling location N2)

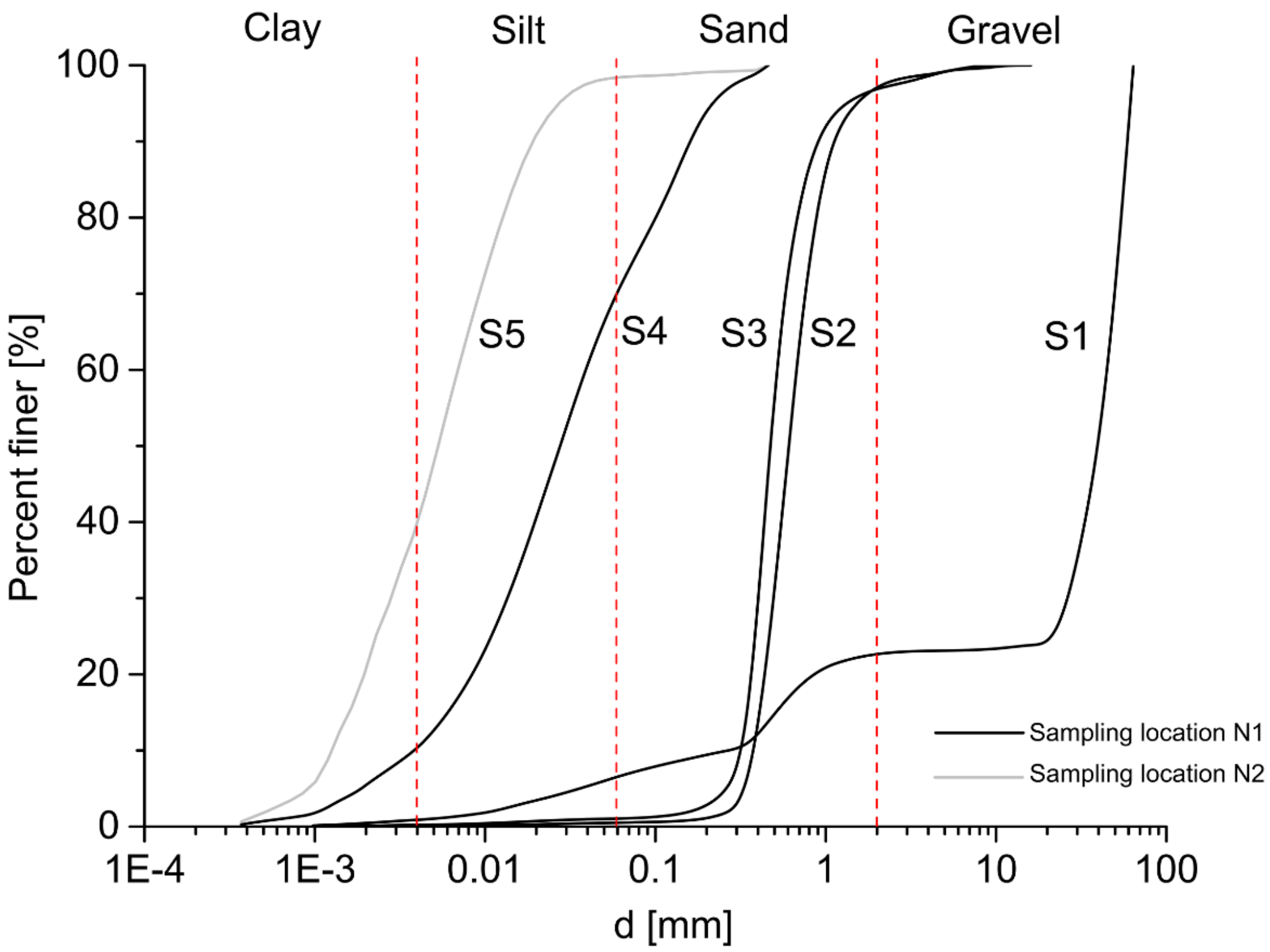

Fig. S2 Grain-size distribution of collected sediments. Cumulative grain-size distribution curves of the riverbed sediment samples collected from the Arno River (sampling location N1 and N2 in black and gray, respectively). Red dash lines represent different size classes (clay, silt, sand and gravel)

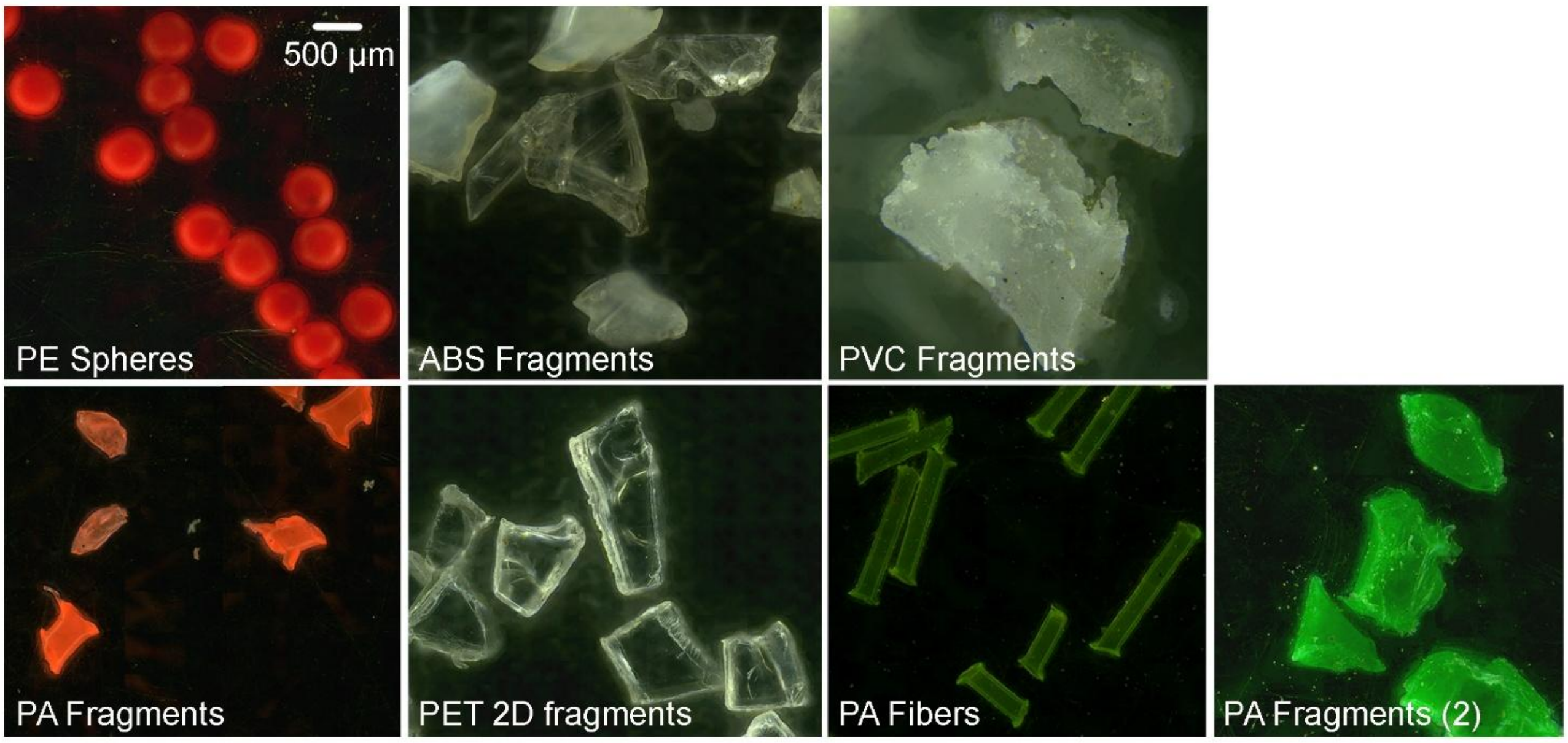

Fig. S3 Reference MPs used for method validation. Optical microscopy images of reference MPs encompassing different polymer types, shapes, and sizes to simulate environmental contamination (see **Table S1**): polyethylene (PE, 0.98 g/cm^3) spheres and 2D fragments, acrylonitrile butadiene styrene (ABS, 1.01 g/cm^3) fragments, polyvinyl chloride (PVC, 1.084 g/cm^3) fragments and polyamide (PA, 1.14 g/cm^3) fragments and fibers

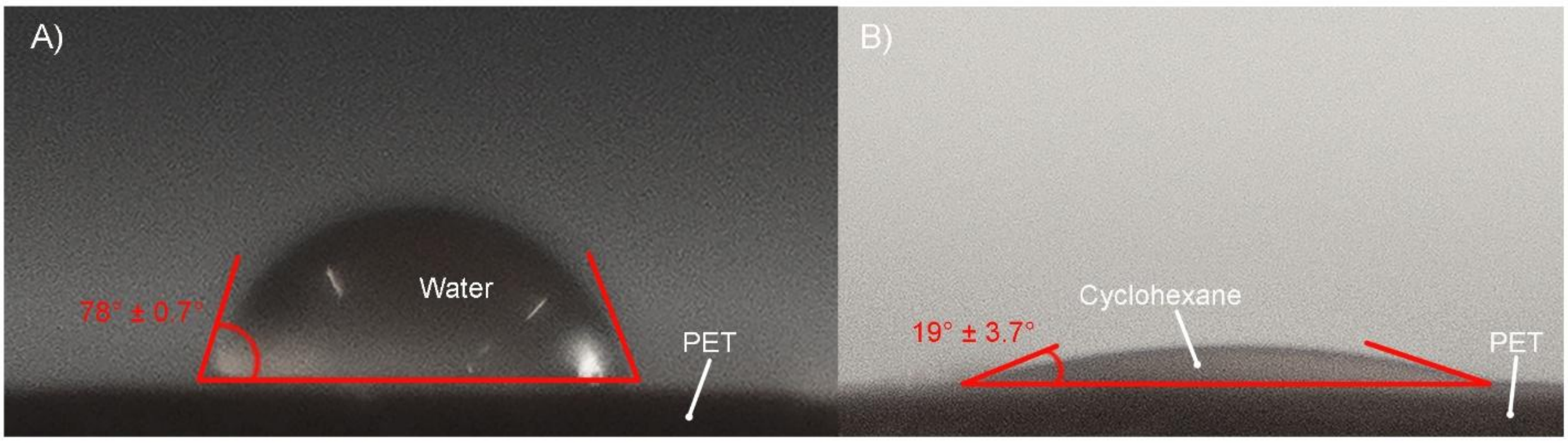

Fig. S4 Contact angle measurements on PET. (A) Water and (B) cyclohexane droplets were dispensed using a precision pipette with a volume of 10 µL. Contact angle values were determined using the ContactAngle tool in ImageJ and represent the average of five measurements for each liquid

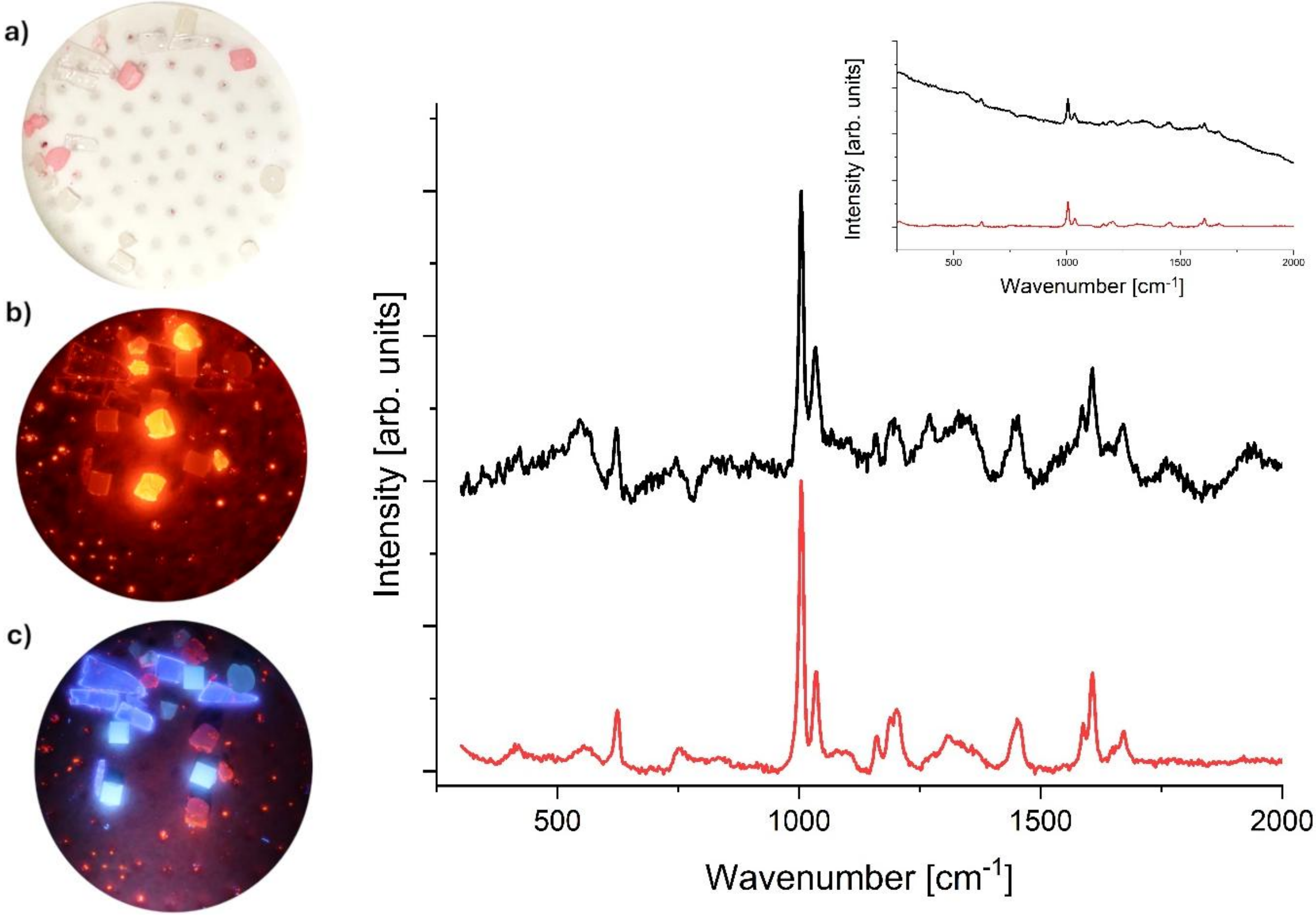

Fig. S5 Effect of oxidation and staining on microplastic fluorescence. Left column: Imagines of reference particles (PVC and ABS) treated with Nile red staining under (a) standard RGB illumination and fluorescence with (b) 532 nm excitation (590 nm cutoff filter in detection) or (c) 370 nm excitation (UV filter in detection). Right graphs: Baselined Raman spectra with 785 nm excitation of reference ABS particle dropped in cyclohexane and let dry in air (bottom, red trace) and ABS particles Nile Red stained after Fenton oxidation (top, black trace). The same experimental conditions were used. The spectra were normalized to the intensity of the main peak and vertically shifted for better visualization. In the inset the raw data are shown.

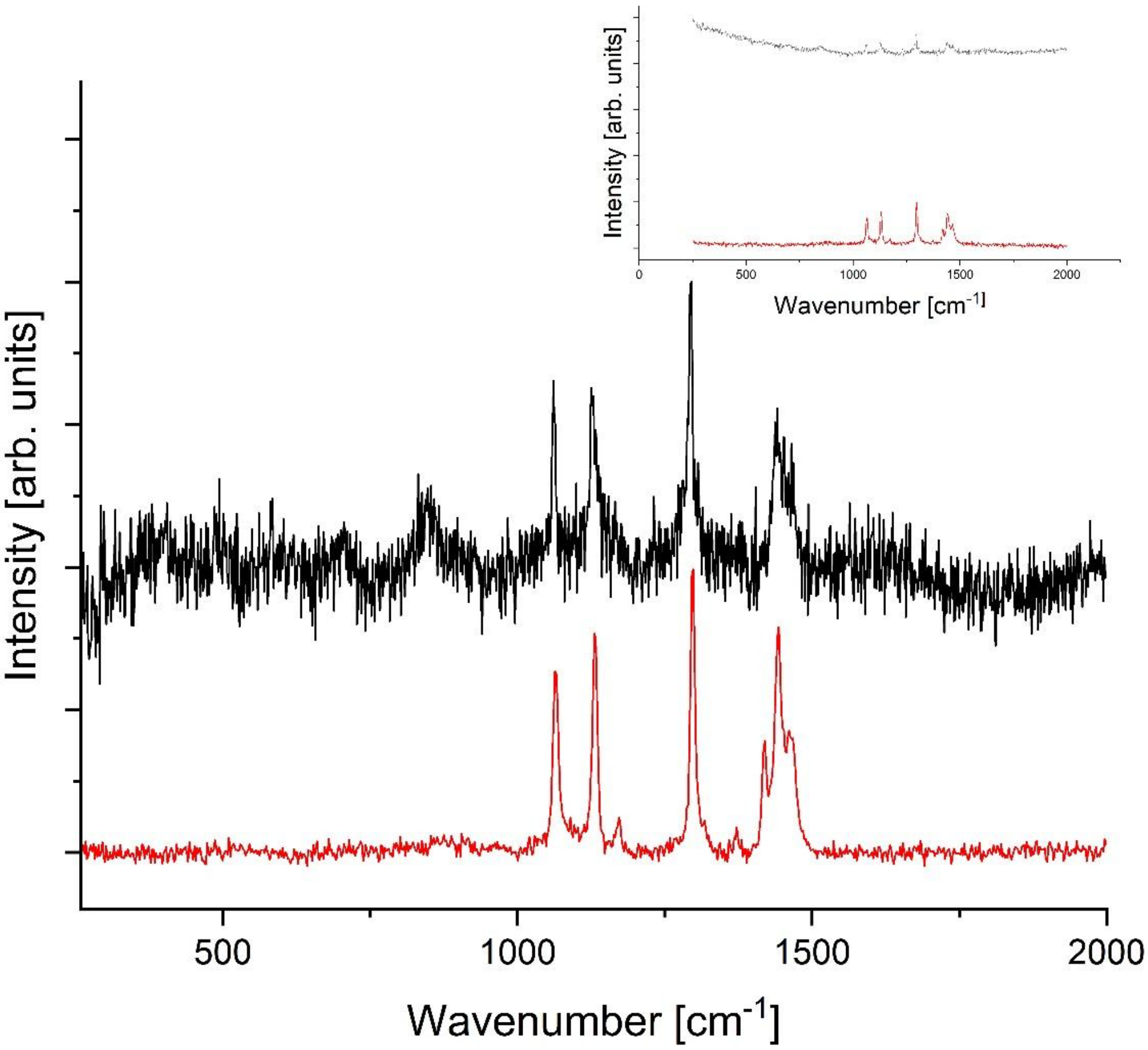

Fig. S6 Raman interference from olive oil residues. Raman spectra with 785 nm excitation of reference PE particle dropped in cyclohexane and let dry in air (bottom, red trace) and PE particle extracted with the olive oil method, washed 3 times with cyclohexane and let dry in air (top, black trace). The same experimental conditions were used. The spectra were normalized to the intensity of the main peak and vertically shifted for better visualization. In the inset the raw data are shown.

Table S1. Physical and chemical properties of reference MPs. Density, size (expressed as the nominal diameter), shape, aspect ratio and contact angle with water of the different reference MPs used in this paper (**Figure S3**). The particle sizes are evaluated using ImageJ on an average of twenty MPs. The contact angles with water are taken from the literature for pristine materials ([a] taken from Ref. (Ellison and Zisman 1954),[b] taken from Ref. (Zhang et al. 2025),[c] taken from Ref. (Scolaro 2019)).

Reference MPs	*Density (g/cm^3)*	*D_n (μm)*	*Shape*	*Aspect ratio*	*Contact angle with water (°)*
PE spheres	0.98	388.1 ± 30.7	Spheres	1.02	95[c]
ABS fragments	1.01	677.6 ± 304.4	Fragments	1.62	82[b]
PVC fragments	1.084	1609.2 ± 685.1	Fragments	1.80	85[b]
PA fragments	1.14	571.4 ± 95.9	Fragments	1.77	70[a]
PA fragments (2)	1.14	744.9 ± 191.6	Fragments	1.71	70[a]
PA fibers	1.14	448.7 ± 68.4	Fibers	5.26	70[a]
PET 2D fragments	1.30	1747.1 ± 419.1	2D fragments	1.62	81[a]

Table S2. Detailed recovery fractions for all efficiency tests. MP recovery fraction for polyethylene (PE), polyvinyl chloride (PVC) acrylonitrile-butadiene styrene (ABS), polyamide (PA) and polyethylene terephthalate (PET) for the cyclohexane extraction process, for the flotation process, and the overall process tested in different sediment samples or with different shapes. Also, the Figure on which the data are represented is indicated.

Flotation and overall process efficiency [Recovery rate] (Figure 3)						
	Flotation efficiency [Recovery rate]			*Overall efficiency [Recovery rate]*		
Granulometry	*PE*	*PVC*	*PET*	*PE*	*PVC*	*PET*
**Coarse gravel**	0.88 ± 0.06	0.87	0.40 ± 0.06	0.83 ± 0.07	0.79	0.36 ± 0.06
**Coarse sand**	0.96	0.95	0.51	0.92	0.86	0.46
**Medium sand**	0.93 ± 0.06	0.76	0.52	0.88 ± 0.06	0.69	0.47
**Fine sand**	0.77	0.69	0.42	0.74	0.63	0.38
**Coarse silt**	0.80	0.75	0.44	0.76	0.68	0.40
**Fine/medium sand**	0.76	0.75	0.47	0.72	0.68	0.42

Cyclohexane and overall process efficiency [Recovery rate] (Figure 4)		
Reference MPs	Cyclohexane efficiency	Overall process efficiency
**PE spheres**	0.95 ± 0.04	0.85 ± 0.02
**ABS fragments**	0.93 ± 0.05	0.75 ± 0.07
**PVC fragments**	0.91 ± 0.07	0.63 ± 0.06
**PA fragments**	0.80 ± 0.04	0.72 ± 0.03

**PA fragments (2)**	0.83 ± 0.06	0.75 ± 0.05
**PA fibers**	0.75 ± 0.07	0.61 ± 0.14
**PET 2D fragments**	0.90 ± 0.05	0.47 ± 0.07

Table S3. Environmental MP concentrations and polymer types. MPs concentration, expressed as items of MP over grams of sediments (g.s.), and polymers found in the six different environmental samples.

ID_Sample	*MPs items/g.s.*	*Polymer types*
S1	0.210	PP, PES, PE
S2	0.092	PES
S3	0.065	PE
S4	0.065	PES
S5	0.249	PES, PP, PE
S6	0.653	PE, PC, PET, PES

References

Ellison AH, Zisman WA (1954) Wettability Studies on Nylon, Polyethylene Terephthalate and Polystyrene. J Phys Chem 58:503–506. https://doi.org/10.1021/j150516a013

Scolaro C (2019) Progettazione di materiali innovativi a base di polietilene per applicazioni ingegneristiche avanzate. PhD thesis

Scopetani C, Chelazzi D, Mikola J, et al (2020) Olive oil-based method for the extraction, quantification and identification of microplastics in soil and compost samples. Science of The Total Environment 733:139338. https://doi.org/10.1016/j.scitotenv.2020.139338

Zhang Y, Zhao Y, Qian Z, et al (2025) A hydrophilic/hydrophobic switch on polymer surface triggered by calcite towards separation of hazardous PVC from plastic mixtures. J Hazard Mater 483:136667. https://doi.org/10.1016/j.jhazmat.2024.136667
